# An International Survey of Cyanobacteria Risk Management in Recreational Waters: Methods, Principal Taxa and Toxins, Challenges and Perspectives

**DOI:** 10.1007/s00267-026-02577-z

**Published:** 2026-08-06

**Authors:** Daniel J. Franklin, Laura T. Kelly, Diane M. Orihel, Jonathan Puddick, Susanna A. Wood

**Affiliations:** 1 School of Life & Environmental Sciences, Bournemouth University, Poole, Dorset UK; 2 Cawthron Institute, Nelson, New Zealand; 3 Department of Biology and School of Environmental Studies, Queen’s University, Kingston, ON Canada; 4 Department of Environmental Management, Lincoln University, Christchurch, New Zealand

## Abstract

World Health Organization (WHO) guidance on the management of cyanobacteria in recreational waters provides a comprehensive framework for reducing human health risks. To provide a global overview of how cyanobacterial risks in recreational waters are managed and to better understand capacity gaps and best practice, we surveyed expert opinion across the world. A total of 161 survey responses were collected from 49 countries. Results indicate widespread variation in the extent of recreational water monitoring. Routine monitoring often varies by region in larger, more populous countries, while seasonal monitoring, typically conducted at variable intervals, was the most used approach. Approximately half of the countries reported either a lack of routine monitoring or a combination of different monitoring patterns. Notably, whereas nearly all countries reported methods for planktonic sampling, only one-third of countries reported methods for benthic sampling. Globally, we conclude that the assessment and management of benthic cyanobacteria risks lag behind that of planktonic cyanobacteria risks. Cell counts and pigments were the most common assessment techniques for planktonic cyanobacteria. *Microcystis* and *Oscillatoria* were the most frequently reported problematic genus of planktonic and benthic cyanobacteria, respectively, and microcystins were the most frequently reported cyanotoxins of concern. A wide variety of management responses and risk communication methods were reported with the primary action trigger being the presence of cyanobacterial scums. Experts across the world were mostly unsatisfied with recreational water risk management regimes, with over 80% of respondents desiring change in how cyanobacterial risks are managed. We conclude that a renewed international effort is needed to enhance collaboration and knowledge exchange and sustain investment to ensure safer recreational waters at a global scale.

## Introduction

Harmful cyanobacterial blooms occur in freshwater and coastal ecosystems worldwide. For example, *Microcystis*—one of the most pervasive bloom-forming cyanobacteria in freshwater ecosystems—has been reported in 108 countries, and on all continents except Antarctica (Harke et al. 2016). In addition to *Microcystis*, other toxin-producing cyanobacterial genera commonly reported on a global scale include *Dolichospermum* (previously *Anabaena*)*, Aphanizomenon, Planktothrix* and *Oscillatoria* (Svircev et al. 2019). While harmful cyanobacterial blooms are most often associated with massive surface accumulations of planktonic cyanobacteria, many reports of benthic toxin-producing cyanobacteria have emerged over the last two decades. Proliferations of benthic toxin-producing cyanobacteria, such as *Anabaena, Nostoc, Oscillatoria and Microcoleus* (*Kamptonema/Phormidium*), have been documented in streams, rivers, lakes and ponds in many countries (Wood et al. 2020).

Cyanotoxins are a significant public health issue, leading to adverse health effects in humans that range from minor contact irritation and gastrointestinal distress to lethal poisonings (Carmichael and Boyer 2016). Cyanotoxins are a broad suite of chemical compounds, including cyclic peptides (e.g., microcystins and nodularins), alkaloids (e.g., anatoxins, saxitoxins, cylindrospermopsin, aplysiatoxin and lyngbiatoxin-a) and lipopolysaccharides, among others (Rastogi et al. 2015). People are exposed to cyanotoxins via three pathways: consumption of contaminated food (e.g., fish, crops and food supplements), ingestion of contaminated drinking water, and through skin contact, inhalation and accidental ingestion during recreational activity (Merel et al. 2013). The potential for recreational exposure may be increasing in some locations; e.g., in the United Kingdom, public demand for increased recreational opportunities in freshwaters has recently resulted in the designation of many more official “inland bathing” sites (Spurr et al. 2025). At the same time, eutrophication and climate change are increasingly recognized as drivers of the rising prevalence of harmful cyanobacterial blooms. Climate change contributes through factors such as elevated water temperatures (Paerl and Huisman 2009; Woolway et al. 2021) and enhanced internal nutrient loading driven by changes in lake stratification (Orihel et al. 2017).

Since the 1990’s, the World Health Organization (WHO) has provided guidance on the risks associated with cyanobacterial blooms in recreational waters and strategies to manage the risk (Chorus et al. 2010). The WHO recreational water advice was updated in 2021 (World Health Organization 2021) and recommends implementing a three-level monitoring and action framework (with Vigilance, Alert 1 and Alert 2 states) to protect public health. At the same time, the WHO guidance recommends the development of specific national approaches given the large global variation in cyanobacteria species, toxins, government resources and expertise, hydrology, and other social factors (e.g., risk appetite). Therefore, recreational cyanobacterial water risk management is likely to vary significantly between countries and even within larger countries. With so much variation, we suggest that assessments of how different countries and regions are managing cyanobacteria risks are useful to inform future national and international policy development and risk management regimes. They also have the potential to provide peer-learning opportunities for the recreational cyanobacterial risk management community.

Building on two previous international comparisons, Chorus (2005) and Chorus (2012), Ibelings et al. (2015) gathered information on the management of all cyanobacteria exposure routes for 17 (mostly highly developed) countries. At that time, most of these countries were adopting two- or three-level alert frameworks for recreational water management, albeit with substantial variation between countries in the thresholds for moving between alert levels. In terms of monitoring methods, relatively simple risk assessment methods were favoured (cell counts), which are most practically achievable and allow a wider, although only inferred, assessment of potential toxicity (Ibelings et al. 2015). During the intervening ten years, we are not aware of any other attempt to survey and better understand how international approaches to recreational cyanobacterial water risk management are developing. However, there have been significant technological advancements and the uptake of new monitoring approaches in some locations, such as in the use of remote sensing for bloom monitoring and the use of molecular techniques to target specific species or genera, or the genes involved in cyanotoxin synthesis (e.g., Carratala et al. 2026). Additionally, new threats have emerged, for example, there are increasing reports of toxic benthic cyanobacteria (Kelly et al. 2026). Benthic cyanobacteria are relatively poorly understood in many locations and require different monitoring and risk assessment approaches.

Against the backdrop of increasing cyanobacterial bloom risks in some regions (IPCC 2022), we aimed to evaluate how recreational cyanobacterial water risk management varies and to better understand the principal issues facing water managers and public health scientists. To do this, we developed an online survey that was distributed and promoted via multiple channels. By targeting global experts who work in the field we sought to:

1. Quantify global variation in national and regional approaches to recreational cyanobacterial monitoring.
2. Assess and rank the principal cyanobacterial taxa and toxins of global concern.
3. Evaluate how management action thresholds and public risk communication vary globally.
4. Collate perceptions of the adequacy of cyanobacteria risk management and opinions on how management regimes could be improved.

## Methods

### Survey Design and Deployment

We designed an online survey (JISC online surveys - v3), which was granted ethical approval (Bournemouth University ethics ID 56031). Participants were provided with a participant information sheet and gave consent before proceeding. Participants could remain anonymous but were required to define their location (country and subnational region, if appropriate) and level of confidence, as assessed by the question: “How confident are you in describing how cyanobacteria risks are assessed and communicated?”. Questions then proceeded in two topic sets: 1) risk management, including cyanobacteria monitoring regime, cyanobacteria assessment methods, habitat types assessed, problematic taxa and toxins, and thresholds for action, and 2) risk communication, methods and water body interventions, opinions on the adequacy of current management and communication, and suggestions for improvements in the management regime. The complete set of survey questions can be viewed in the Supplementary Material Section S1.

### Participant Recruitment and Data Collection

The survey was open for five months between February and June 2025. Members of professional interest groups (e.g., Algae-L, the UK Harmful Algal Network, Society of Canadian Aquatic Sciences) were directly invited to participate, as were a list of professionals selected based on their public contributions (e.g., publications, reports, work in government environmental or safety agencies) to this research, policy and management area. The survey was also publicised at the 13th International Conference on Toxic Cyanobacteria (ICTC13) conference in Chania (Greece) in early June 2025 and some additional invitations were sent to potential participants in countries/continents with low response rates towards the end of the survey period.

### Post Survey Data Processing and Analysis

Participants who self-identified as not confident (6% of respondents) were excluded from the subsequent analysis (excepting the qualitative analysis of opinion responses). Survey responses were then summarised. Where results are displayed using regional groupings, the data is presented as a percentage of responding countries to reduce the bias from an uneven distribution of survey responses (or countries) in a region and between regions. Where it was given, the email address of the survey respondents was used to classify the respondents professional background using the following categories: academic/research (university), academic/research (non-university), government, consultancy, and other.

Country representation of responses was calculated from the number of respondents who selected each country. These were binned into categorical groups to aid visualisation, with bins of 0, 1–2, 3–6, 7–10 and >10. Responses to the question about routine monitoring were grouped by response (yes, no, unsure and no-response). To facilitate reporting by country, responses were coalesced when there were multiple responses that differed within a country. If all responses from a country were ‘yes’, the monitoring was designated as “routine”. If both ‘yes’ and ‘no’ responses were received from the same country, the monitoring was designated as “mixed”. If only ‘no’ responses were received, the monitoring was designated as “none”. Two fallback cases were included if a country had only ‘unsure’ or ‘no response’ results, which were designated as “unclear” and “no response”, respectively.

A similar coalescing strategy was applied to the type of monitoring and the habitats being monitored. If all responses within a country indicated “only planktonic types are assessed”, the result was designated as “planktonic only”. Where any responses from a country indicated that “both are assessed”, the country was designated as “both benthic and planktonic are assessed”. Likewise, if all responses indicated only lakes are monitored, the response was designated “lakes only”. If any respondents indicated both lakes and rivers were monitored, then the response was designated as “lakes and rivers”, with no responses left as “no response”.

Processing of data for questions relating to the taxa that form blooms or cause problems was undertaken in two steps. The first extracted the values from the selection boxes of previously defined taxa available in the survey. The second step required visual assessment of the free-text category to identify taxon matches and keywords that related to specific taxa. These were then appended to the list of taxa from the pre-selected list to obtain a full list of taxa that respondents had identified. Due to taxonomic revisions, we coalesced some taxa for display purposes, including *Oscillatoria* and *Oscillatoria* spp., and *Anabaena* and *Dolichospermum* for the planktonic taxa list. For the benthic taxa list, *Microcoleus* and *Phormidium* were coalesced, as were *Microseira* and *Lyngbya*. Where coalescing has occurred, this is indicated in subsequent analyses using an asterisk (*).

Processing of data for questions 21/22, 23/24 and 26/27 relating to action triggers for public health warnings and risk communication mechanisms was undertaken in two steps. First, data was extracted from the pre-defined selection boxes in the primary question (i.e., questions 21, 23 and 26). Then a manual assessment of the free-text question (i.e., questions 22, 24 and 27) was used to identify additional methods used to trigger health warnings or for risk communication and the number of responses from each country was tallied. When methods weren’t selected in the primary question with pre-defined options but were mentioned in the free-text question, these were added to the tally for the relevant category.

Questions 12 and 28 provided free-text data on monitoring frequencies and risk management interventions. A manual assessment of the responses was used to identify suitable categories and the number of responses from each country was tallied.

## Results

### Survey Responses: Geographic Representation and Respondent Type

A total of 161 survey responses were received from participants in 49 countries. Of the 161 respondents, we could classify the professional background of 83%. The largest professional category of respondents was academic/research (university) at 40%, followed by government (20%), then academic/research (non-university) at 12%, other (7%), and consultancy (4%). Ten respondents self-identified as “not confident”, in describing how cyanobacteria risks are assessed and communicated, and these responses were removed for all subsequent analyses, except for qualitative assessment of opinions. Of the remaining respondents, 92 self-identified as “very confident”, 56 as “somewhat confident” and the remaining three did not answer this question. Following this filtering step, 46 countries remained represented in the dataset for subsequent analyses.

**Fig. 1:**
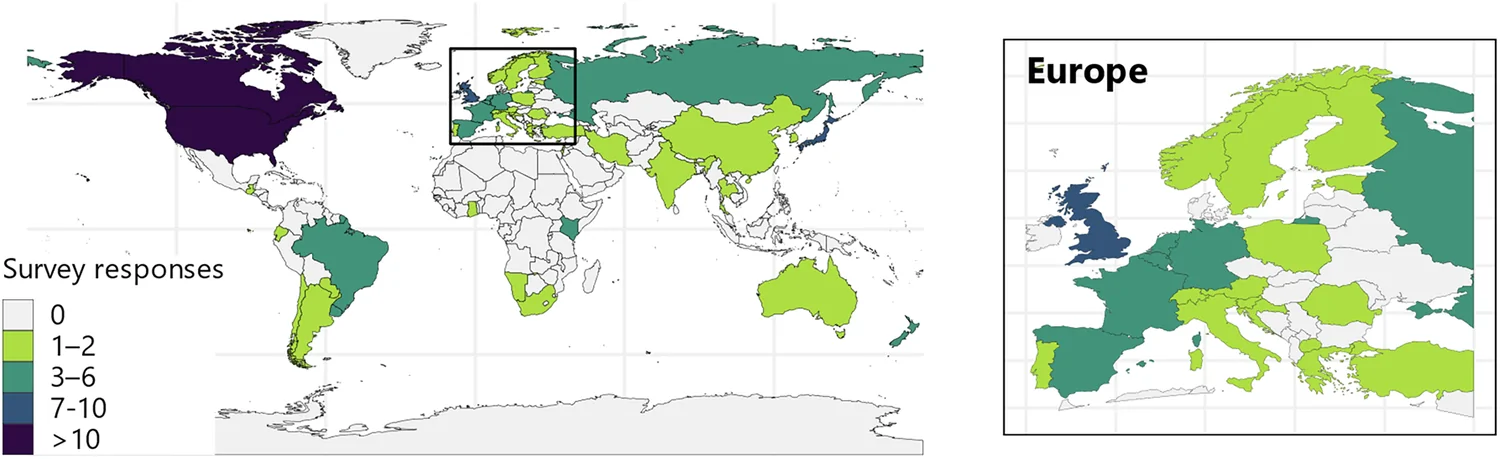
Geographic distribution of the number of survey respondents per country, excluding those who self-selected as “not confident” in describing how cyanobacteria risks are assessed and communicated

Responses were geographically diverse (Fig. 1), with representation from Africa (7 responses), Asia (18 responses), Europe (57 responses), North America (52 responses), Oceania (5 responses) and South America (13 responses).

### Recreational Water Risk Management: Monitoring and Type of Cyanobacteria Assessed

**Fig. 2:**
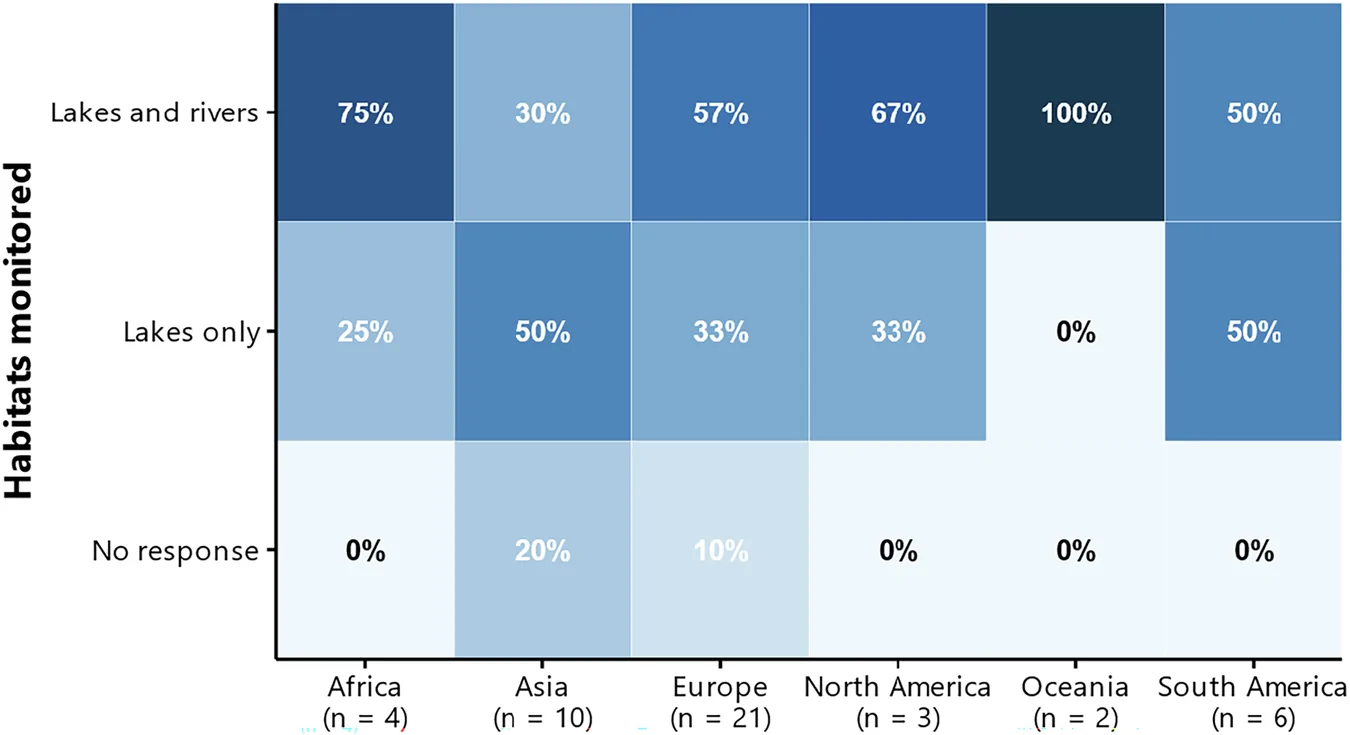
Proportion of countries in each region that report the monitoring of both lakes and rivers, lakes only or did not respond to the question. The n below the region name is the total number of countries represented in that region. No respondents chose rivers only

Across regions, most respondents reported that routine monitoring at their location (defined in the survey as assessments that occur before obvious blooms are evident) occurred in both lake and river habitats (Fig. 2) with the highest proportion of lake and river monitoring occurring in Oceania (100%) and North America (67%), followed by Africa (75%) and Europe (57%). Monitoring of only lakes was most common in Asia (50%) and South America (50%).

**Fig. 3:**
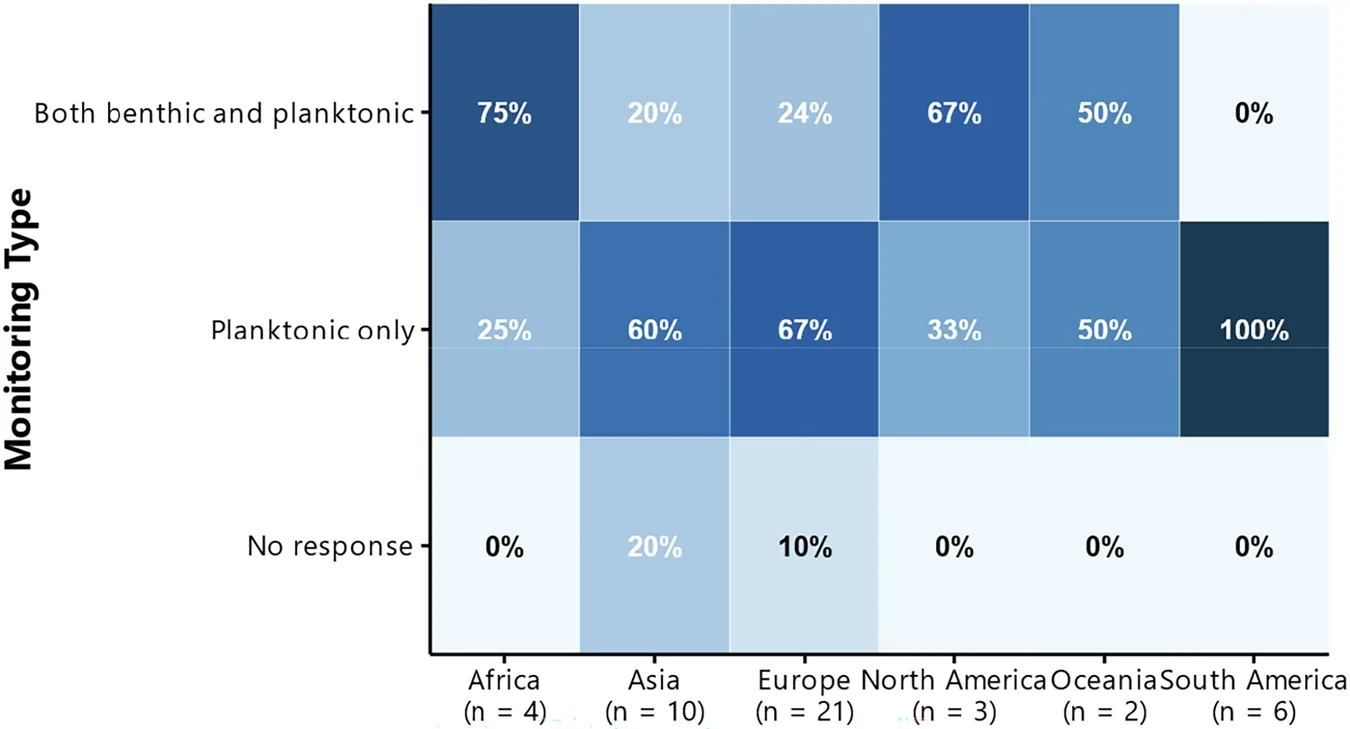
Proportion of countries in each region that report routine monitoring for both benthic and planktonic cyanobacteria, planktonic cyanobacteria only or did not respond to the question. The *n* below the region name is the total number of countries represented in that region. No respondents chose benthic only

Most regions reported routine monitoring for planktonic cyanobacteria only (Fig. 3), with the highest proportions of planktonic only monitoring in South America (100%), Europe (67%), and Asia (60%). Monitoring for both benthic and planktonic cyanobacteria was reported as most common in Africa (75%) and North America (67%), with Oceania at 50% and no countries in South America reporting this type of monitoring. Non-response was low overall (≤20%) and we noted that selection of this option likely indicates no monitoring activity.

### Use of Routine Cyanobacteria Monitoring

**Fig. 4:**
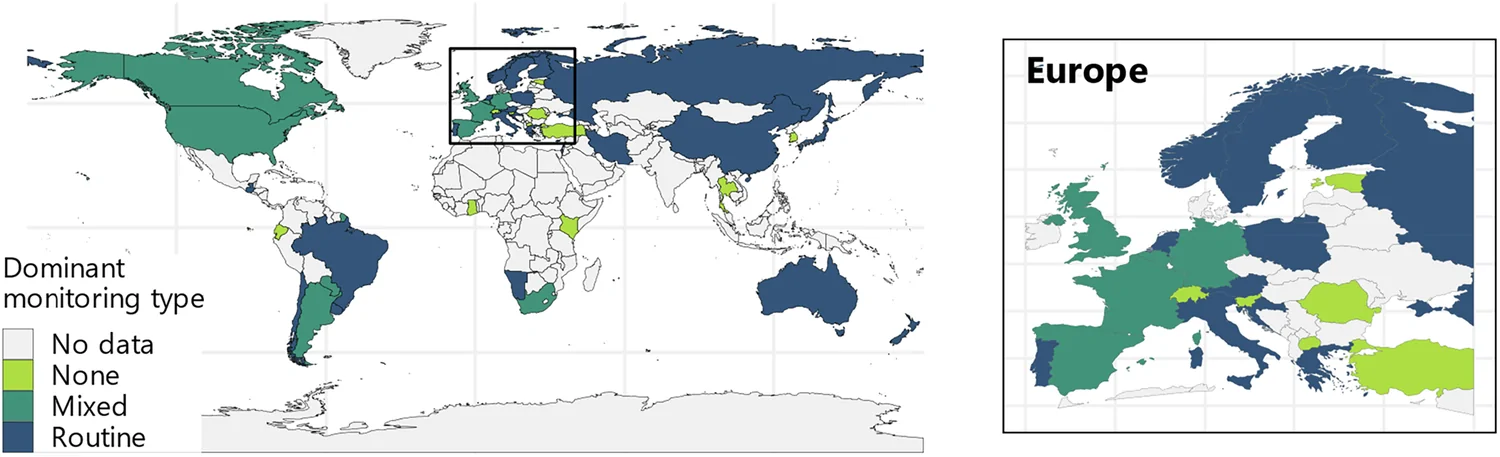
Monitoring strategies for cyanobacteria reported by country, where routine indicates there is proactive monitoring (monitoring in the absence of visible cyanobacteria), mixed indicates both routine and no monitoring responses reported from that country and none indicates all respondents identified no routine monitoring

Routine monitoring (defined in the survey as proactive assessments that occur before obvious blooms are evident) was most common in Europe, where eleven countries reported routine monitoring, followed by Asia (6) and South America (3; Fig. 4). Mixed responses, indicating variability within countries, were reported primarily in Europe (5) and Asia (5), with smaller numbers in North and South America (2 each).

### Frequency of Cyanobacteria Monitoring

Of the participants that responded to Question 12 (How frequently is monitoring for cyanobacteria carried out at your location?), responses were wide ranging (Supplementary Material S2). In larger countries with federal systems, such as Canada and USA, monitoring frequency was region-specific (e.g. at the provincial or state level, respectively). In smaller countries there was more continuity in responses, although several different monitoring frequencies were suggested for multiple countries. Some respondents (19) misinterpreted the question and provided information on when monitoring was undertaken (rather than the monitoring frequency), triggers for health warnings, monitoring locations, or monitoring frequencies for drinking-water situations.

Seasonally variable monitoring frequencies were more common than consistent year-round monitoring frequencies. The most adopted year-round monitoring frequency was quarterly (four-times per year; 6 responses), then monthly (twelve-times per year; 4 responses), then annually (once per year; 3 responses). During the ‘bloom season’ (the seasons when cyanobacterial blooms are more likely; spring/summer/autumn), there was a relatively even spread in monitoring frequencies between weekly (15 countries), fortnightly (13 countries), monthly (19 countries) and responsive monitoring (15 countries). During winter, when cyanobacterial blooms are less likely to occur, monthly monitoring was the most common response among countries. Some respondents also noted that there was no monitoring undertaken for recreational public health purposes in their country and that the only cyanobacterial monitoring that occurred was for research purposes.

Multi-tier risk management approaches, with escalating monitoring frequencies were adopted by multiple countries, e.g., in France, Italy, the Netherlands, New Zealand, Spain, and USA. Likelihood for human exposure also guided the monitoring frequency adopted in some countries, with some popular swimming beaches undertaking sampling twice a week during summer and daily visual checks for cyanobacteria. In some countries, routine monitoring is undertaken in the lead-up to water-sports events being undertaken at waterbodies where cyanobacterial blooms regularly occur. In New Zealand, where national recreational cyanobacteria guidelines have been established for over 15 years, monitoring frequency appeared to also be nuanced by local knowledge, with some regions undertaking weekly monitoring for benthic cyanobacteria in streams and/or rivers during the summer despite the recommended frequency in the guidelines being fortnightly (Ministry for the Environment and Health New Zealand 2024).

### **Cyanobacteria Assessment/Sampling Methods**

**Fig. 5:**
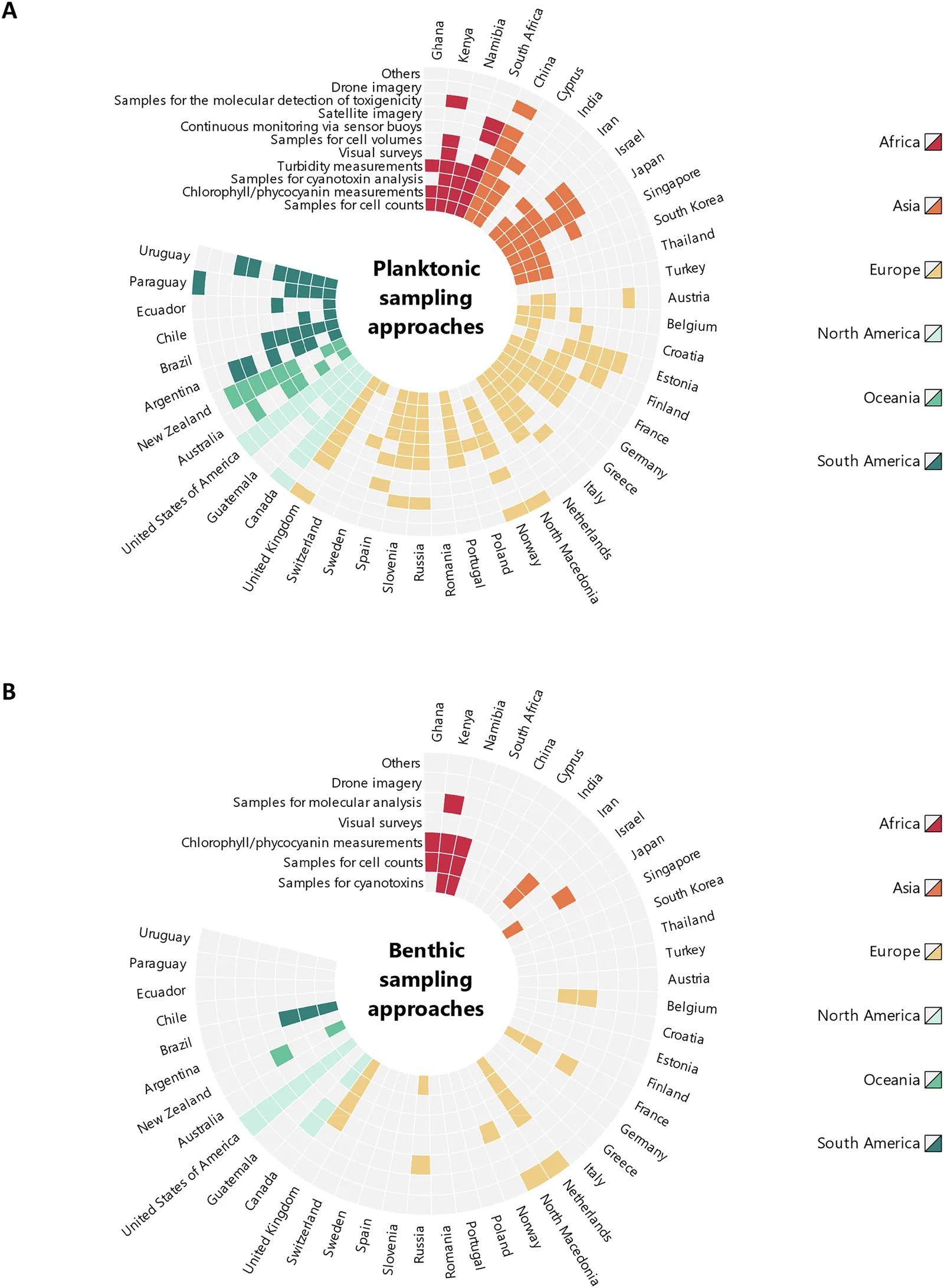
Sampling approaches employed in each country for planktonic cyanobacteria (**A**) and benthic cyanobacteria (**B**). Coloured cells indicate the monitoring method is reported as used in that country, whilst light grey indicates the method is not used. Methods are ordered from most common across countries in the centre to least common at the periphery

The most common sampling method for planktonic cyanobacteria was the collection of water samples for cell counts, followed by analysis of cyanobacterial pigments as a proxy for abundance (Fig. 5A). Despite the growing interest in remote sensing, satellite and drone imagery were not reported to be widely used. Taking samples for cyanotoxin analysis was the third most widely used method, whilst molecular detection of toxigenicity was one of the least widespread sampling approaches. For benthic cyanobacteria, samples for cyanotoxins and cell counts were the most widely employed methods (Fig. 5B). Since benthic cyanobacteria were reported as not sampled in many of the survey responses (i.e., only 16 of 46 countries reported benthic sampling; Fig. 3) the finding of fewer benthic sampling techniques is perhaps also unsurprising, and contrasts strongly with the wide array of sampling approaches undertaken globally for planktonic taxa. The USA was notable as being the only country reporting the use of all planktonic and benthic sampling techniques.

### Problematic Cyanobacteria Taxa and Toxins in Recreational Waters

**Fig. 6:**
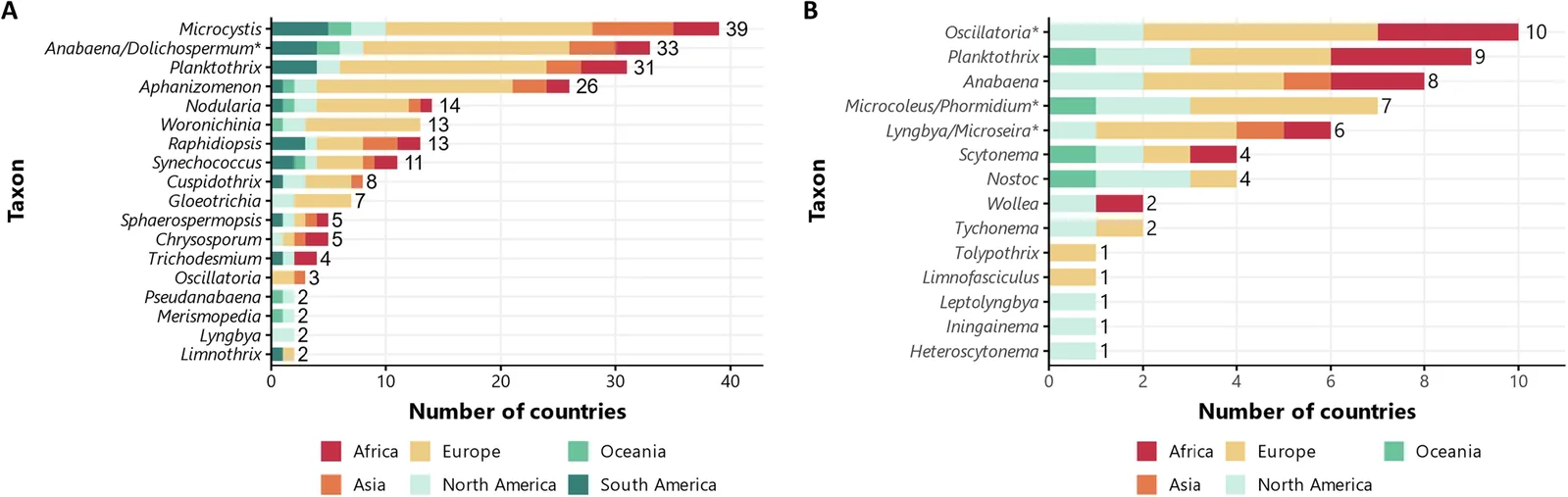
Taxa reported as causing blooms or proliferations by survey respondents in planktonic environments (**A**) and benthic environments (**B**). Note that Anabaena and Dolischospermum are combined for this as taxonomic nomenclature changes mean they are likely the same taxonomic group. Likewise, Microcoleus and Phormidium, and Microseira/Lyngbya have been combined, and *Oscillatoria* includes cases where respondents specified *Oscillatoria* spp

*Microcystis* was the most frequently reported planktonic bloom-forming taxon (39 countries), followed by *Dolichospermum* (33) and *Planktothrix* (31; Fig. 6A). Other notable taxa included *Aphanizomenon* (26), *Nodularia* (14), *Woronichinia* and *Raphidiopsis* (13 each). *Synechococcus* was reported by eleven countries, which was unexpected given its typically non-bloom-forming role. For benthic taxa, *Oscillatoria* was the most reported benthic taxon (10), followed by *Planktothrix* (9) and *Anabaena* (8). Other benthic-associated genera such as *Microcoleus, Microseira*, and *Pseudanabaena* were reported less frequently (Fig. 6B).

**Fig. 7:**
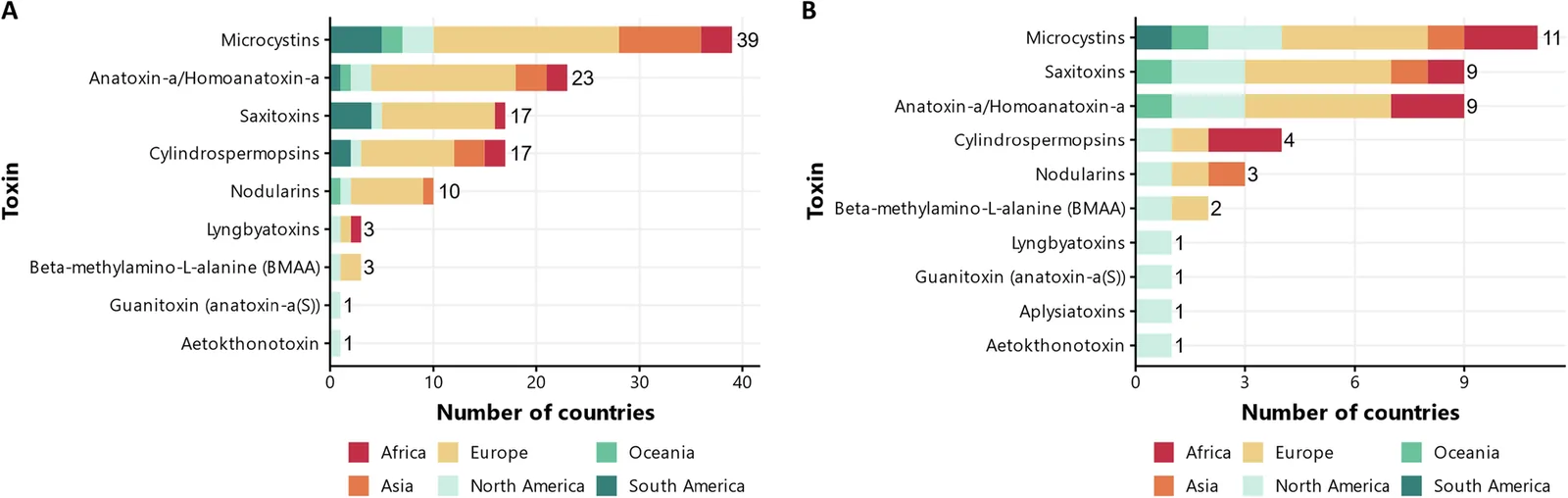
Number of countries in which each class of toxin was reported as causing issues or being detected for planktonic cyanobacteria (**A**) and benthic cyanobacteria (**B**). Note the different scales on the x axes and order of toxins on the y axis. The number to the right of the bars is the number of countries

**Fig. 8:**
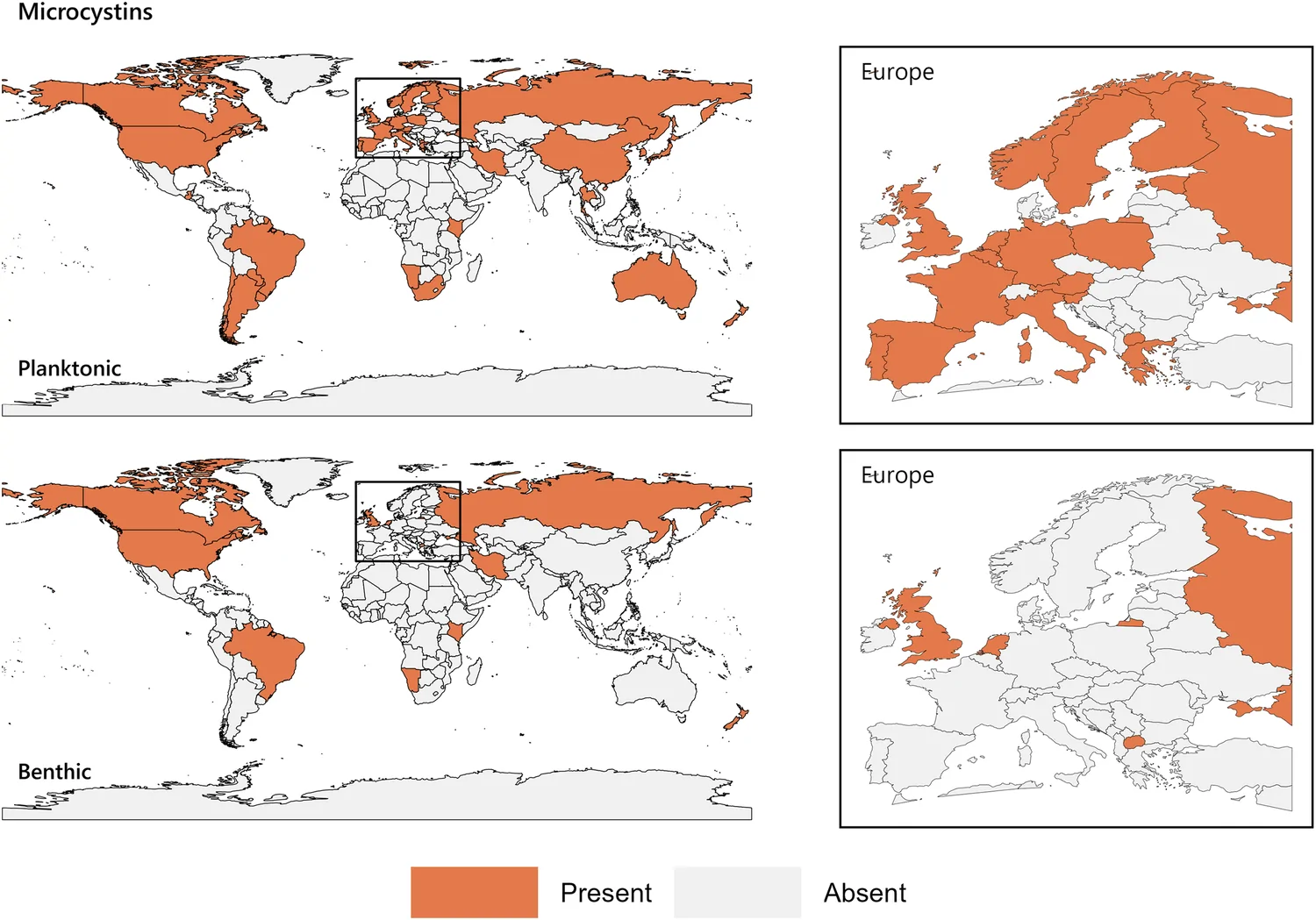
Countries for which microcystins were identified by respondents as posing a risk from both planktonic (top) and benthic (bottom) cyanobacteria. Maps for the other reported toxins (reported from at least two countries) for both benthic and planktonic habitats are also available in Supplementary Materials S3–S7

Microcystins were the most frequently reported toxin produced by planktonic cyanobacteria (39 countries) followed by anatoxin-a/homoanatoxin-a (23) and saxitoxins and cylindrospermopsins (17; Fig. 7A). Guanitoxin/anatoxin-a(S) and aetokthonotoxin were rarely reported (1). Microcystins were also the most frequently reported toxin in benthic environments (11 countries), followed by saxitoxins and anatoxin-a/homoanatoxin-a (9 each; Fig. 7B). Globally, microcystins from planktonic cyanobacteria were more widely reported relative to microcystins arising from benthic cyanobacteria (Fig. 8). Additional reported toxin distribution maps are provided in Supplementary Materials S3–S7.

### Thresholds for Management Action in Recreational Waters

Survey results indicated that a range of data sources are being used to trigger action and issue public warnings related to planktonic cyanobacteria (Questions 21/22; Supplementary Material S8). The visual presence of scums was the preeminent trigger for initiating public health warnings for planktonic cyanobacteria (used in 35 countries; Table 1) followed by cell counts, chlorophyll/phycocyanin measurements and cyanotoxin concentrations (28 countries each). Cyanobacterial biovolumes were also applied relatively frequently (18 countries), but to a lower degree than cell counts. Molecular measurements of taxonomy/toxigenicity were only noted as being used in two countries (Canada and USA). Through the additional comments received (Question 22), several respondents noted that an escalating testing approach was used from lower-cost/-specificity methods (e.g., visual observations and chlorophyll-a/phycocyanin fluorescence measurements) to higher-cost/-specificity methods (e.g., cell counts and toxin testing).

**Table 1:** Result types that trigger action (in the form of public health warnings) for planktonic and benthic cyanobacteria

Habitat	Trigger for public health warnings	Number of countries
Planktonic	Presence of scums	35
Planktonic	Chlorophyll/phycocyanin measurements	28
Planktonic	Cyanotoxin concentrations	28
Planktonic	Cyanobacteria cell counts	28
Planktonic	Cyanobacteria biovolumes	18
Planktonic	App-based citizen science measurements/reports	7
Planktonic	Molecular measurements of taxonomy/toxigenicity	2
Planktonic	None—no public warning system operates	8
Planktonic	Other	7
Benthic	% benthic mat cover	7
Benthic	Cyanotoxin concentration	7
Benthic	Molecular measurements	1
Benthic	None—no public warning system operates	7
Benthic	Other	3

Total number of responding countries = 42 for planktonic and 13 for benthic

Reports from the public, whether ad-hoc or through citizen science apps, were used with moderate frequency (3 and 7 countries, respectively; Supplementary Material S8). Through the additional comments received (Question 22), it was noted that whilst public reports/citizen science data would be used to initiate sample collection (by responsible agencies), it would not necessarily trigger a public health warning. Feedback from two countries (Russia and USA) also noted that reports of human illness and/or animal deaths might be used to initiate testing or trigger public health warnings. Additional data sources mentioned by respondents included Secchi disc measurements (2 countries), satellite imagery (2 countries), on-site rapid tests (2 countries) and bad odours (1 country). Responses from eight countries indicated that public health warnings for planktonic cyanobacteria are not issued at their locations.

There were 3.5-times less respondents who provided a response to Question 23/24 relating to public warnings due to benthic cyanobacteria (38 responses compared 142 responses for planktonic cyanobacteria). Percentage mat cover and cyanotoxin concentrations were the most frequently used data sources for triggering action to issue public health warnings (7 countries each; Table 1). Molecular measurements, reports of human illness and/or animal deaths, detached mats, taxonomic screening by microscopy (1 country), and reports from the public were also noted by respondents, but with lower frequency (Supplementary Material S9). As with planktonic cyanobacteria, multiple respondents (7 countries) indicated that public health warnings for benthic cyanobacteria are not issued in their location.

### Risk Communication and Risk Reduction Methods

The most popular risk communication mechanism amongst respondents was through social media (used in 33 countries) and the use of static signs, which might be permanent (14 countries), modifiable (18 countries) and temporary (25 countries; Table 2). The use of educational/public awareness campaigns was also a popular means of communicating on the risk posed by toxic cyanobacteria (used in 21 countries). Other communications platforms mentioned by respondents included posting data/alerts on websites (12 countries), using interactive maps (4 countries) and specialised phone applications (2 countries; Supplementary Material S10). Other methods of responsive communication included traditional media (e.g., newspaper, radio, television; mentioned by respondents from 10 countries), fliers, pamphlets, email and direct communication with high-risk groups (e.g., water-users, dog owners). Some respondents provided hyperlinks to online dashboards/resources to communicate on the risk from toxic cyanobacteria in their regions, these have been collated in Supplementary Material S11.

**Table 2:** Strategies reported to be used for the communication of public health risk from cyanobacteria

Communication strategy/tool	Number of countries
Social media alerts	33
Static signs (temporary)	25
Educations/public awareness campaign	21
Static signs (permanent/modifiable)	18
Static signs (permanent)	14
Other	19

Total number of countries = 39

The most adopted risk management option mentioned by respondents was the closure of waterbodies to limit potential exposure to toxic cyanobacteria (19 countries; Supplementary Material S12). However, responses may have skewed in favour of this because it was used as the example mentioned in the survey question. Respondents from eleven countries also mentioned the use of public health advisories/warnings as a mechanism for limiting exposure. In some instances (e.g., Canada, New Zealand and USA), respondents mentioned that it was not possible to ‘close’ a waterbody and this is why advisories/warnings are used. Other risk minimisation mechanisms mentioned by respondents included removal/reduction of cyanobacterial blooms (6 countries), limiting high-exposure activities (4 countries), stopping events (which would lead to large numbers of people encountering the water; 2 countries) and limiting promotion of the waterbody as a bathing site (1 country). On the contrary, respondents from 18 countries mentioned that no risk management interventions are made at their location.

As expected, a range of responses were received for Question 25 (i.e., if there is a trigger data value or other criterion, for public warnings at your location please give further brief details). For some countries, respondents reported that there was no trigger value in their country, some reported the use of simple visual assessments and others reported the use of triggers reliant on testing data (e.g., cyanobacterial cell concentrations/biovolumes, taxonomic evaluation, toxin concentrations; Supplementary Material S13). Triggers for benthic cyanobacteria utilised mat coverage assessments and the presence of detached mats. Multiple respondents from different continents (e.g., Europe and South America) reported using the trigger values from the WHO guidelines for cyanobacteria. Some respondents opted to supply links to online resources that described trigger values in their region, these have been collated in Supplementary Material S11.

### Opinions on the Adequacy of Cyanobacteria Risk Management and Potentially Useful Changes to the Management Regime

**Fig. 9:**
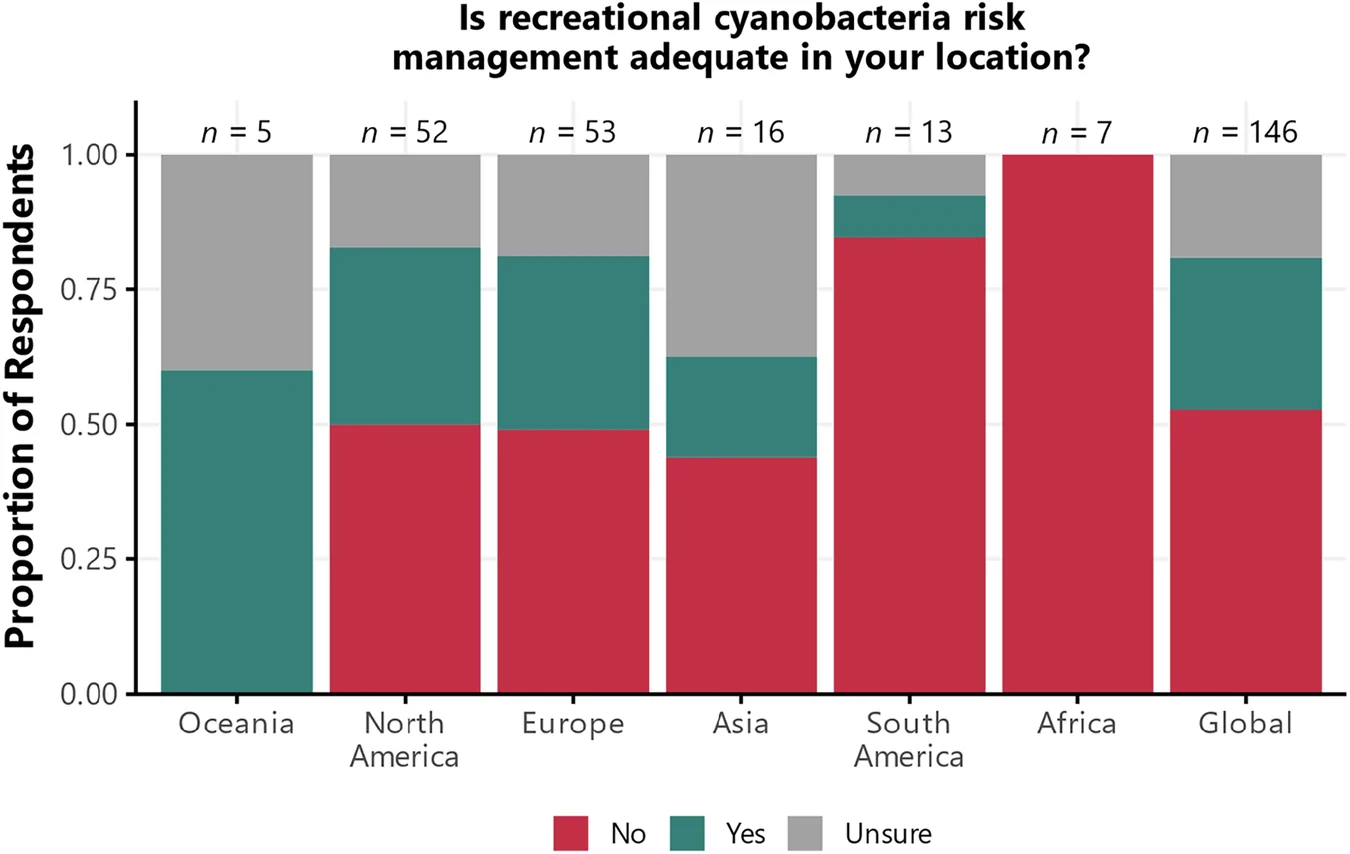
Proportion of survey responses considering whether recreational cyanobacterial risk management was adequate in their location. The global bar combines all responses for this question that were not left blank. The *n* above the bars is the number of responses for each region

At the end of the survey, participants were invited to offer opinions on the adequacy of cyanobacteria risk management at their location and to share ideas on how it could be improved. Most respondents (54%) considered that risk management was not adequate (Fig. 9) with this view most pronounced in Africa and South America, where nearly all responses were negative. Overall, 28% responded that management was adequate with 18% unsure. In terms of participants opinions about the need for changes in how cyanobacteria risk is managed in their location, a large majority (81%) wanted to see changes, whereas only 8% expressed that no change was needed and 11% were unsure of the need for change. Many participants followed up their categorical response with some additional comments, which were further assessed.

Concerns over a) inadequate monitoring, and b) deficient cyanotoxin data were the most frequent type of comment accompanying a negative view of management adequacy. These two concerns were also frequently expressed by those who said they were unsure as to the adequacy of management (18%) at their location. Other substantive issues raised included a damaging lack of national coordination and guidance in some countries, and a concern that monitoring was increasingly relying on independent means (e.g., citizen science and research), which, although clearly a pragmatic response to limited public resources, could be over-relied upon and be poorly designed. Several respondents expressed concern that management was not keeping pace with the increasing risks associated with environmental change, and as expected, respondents from poorer countries frequently cited inadequate resources as a key factor, and in one case, that management efforts were undermined by government corruption.

Of the respondents who thought management was adequate (28%) there was a general view expressed that the systems in place worked well although these respondents also frequently mentioned the issues raised by those who considered management was not adequate or were unsure. Hence, the nature of the concerns over management adequacy were quite consistent amongst respondents. Most respondents (81%) wanted to see changes to the management regime at their location. However, as for the previous question, many of the comments these respondents made were similar in type to those who answered that no changes were needed or who were unsure. Some broad themes were evident in the responses: a desire for better national coordination and action, more rapid, simple and widespread toxin assessment, decentralised monitoring, more and better public involvement and education, more focus on catchment management, and more resources for (better designed) monitoring and warning systems.

## Discussion

### Survey Rationale, Novelty, and Limitations

An international comparison of the management of cyanobacteria in recreational water risks has not, to our knowledge, been attempted for at least 10 years (i.e. since Ibelings et al. 2015). Since that time, the WHO recreational water guidance has been updated (in 2021, superseding the 2003 guidance) and a new IPCC report detailing increasing cyanobacteria impacts and vulnerabilities released (IPCC 2022). Our study is therefore timely and with an extended geographical coverage relative to previous comparisons, including many responses from less developed countries. Most highly populated countries were represented, although there was weaker coverage, when compared to population, in Africa, Asia, Central America and South America. The fact that we had weaker coverage in Central and South America is significant as recent increases in harmful bloom occurrence may be more pronounced in these regions (Feng et al. 2024). Weaker coverage in these areas likely reflects lower monitoring capacity, fewer reported cyanobacterial issues, and challenges in our survey reaching researchers, practitioners and water managers in these regions. It is also worth noting that even in countries with well-developed capacity, management effort and risk may not always be well aligned (Dodds et al. 2023).

It is important to note that this survey is not an exhaustive literature review; for example, the maps of reported toxin distributions and cyanobacteria taxa (and our summaries of the other aspects of our survey) are not a thorough compilation, though they do indicate interesting areas for follow up (See section “Which taxa and toxins are reported as most problematic and where” *below*). In designing the survey, we deliberately tried to limit our requests for personal and professional information reasoning that this would enhance engagement with the survey. Our sense is that, given our survey distribution methods, the survey respondents were typically experienced and competent researchers/practitioners (see also Section “Survey responses: geographic representation and respondent type”) and the majority expressed confidence in their knowledge of the local situation. This work is therefore a wide-ranging review of expert opinion. Of particular interest is the state of opinion amongst this population and their sense of how management could beneficially change in response to the increasing cyanobacteria risks that many respondents identified.

### **What have We Learnt about How Recreational Waters are Monitored?**

Across all regions (except Asia) most countries reported that monitoring occurred in both river and lake systems. Given the WHO 2021 guidance, which advises that blooms do not generally form in “rapidly flowing rivers” and our assumption that the water bodies used for recreation are generally more likely to be natural lakes, or reservoirs/impoundments, this was unexpected. Connected with this finding, it is notable that Asia has a relatively high number of algal bloom modelling studies that are river focused (Murphy et al. 2026) perhaps suggesting a regional preference for modelling over monitoring approaches to river risk management. The WHO advise a water body pre-screening step to assess the likelihood of local environmental conditions to promote blooms, and the result of this assessment should be combined with a parallel assessment of the type and intensity of human use to determine where the three-level alert framework should be implemented. That most countries reported monitoring in both lakes and rivers was somewhat unexpected given the general association of blooms with lakes and that river monitoring is not explicitly mentioned in the WHO guidance. That river-based monitoring is relatively common may indicate both the popularity of rivers for recreation and the increasing importance attached to benthic cyanobacteria risks. There has been a significant increase in river monitoring compared to previous studies (Chorus 2012) and compared to previous reports, there is an increase in benthic monitoring globally, though its absence in South America is notable given the known cyanobacteria issues in that region (Aguilera et al. 2023). Survey responses as to whether planktonic only, or benthic and planktonic types, were assessed during monitoring elicited more mixed responses, and in terms of number of countries, the planktonic only response was dominant. Since benthic cyanobacteria assessments have been formalised into monitoring efforts only recently—firstly by New Zealand (Ibelings et al. 2015)—and with few other countries having yet developed equivalent capacity, this was expected.

Central to risk management in recreational waters is the use of proactive, or routine, monitoring of higher-risk recreational water bodies. Routine monitoring is the cornerstone of effective risk management and higher-risk water bodies should, ideally, be defined through a transparent pre-screening process though this aspect of risk management will vary widely between countries. Our survey responses on whether routine monitoring was implemented were very mixed. Responses were likely influenced by wide-ranging individual interpretations of what was meant by the question and the fact that we had varying responses from within countries (which we designated as a “mixed response”) supports this idea. Our intention was to catalogue official (governmental) efforts, and by extension, citizen science schemes run by researchers which articulate with government regulation and oversight. Given the very mixed responses to this question, we suggest that the understanding of what monitoring is, and what recreational waters are, were very country-specific concepts. In the case of the United Kingdom and the European Union for example, bathing water legislation (the European Union bathing water directive) requires the official designation of bathing water sites and sets out an accompanying obligation for government oversight, meaning a regard for cyanobacterial risks. However, many other non-official recreational water bodies exist, which may be explicitly marketed as such, with the monitoring and risk assessment of those water bodies being much more variable for better or worse.

Despite the expanding awareness of cyanobacterial issues, monitoring and risk management in many countries likely remains largely reactive rather than proactive, suggesting that early-warning systems and preventive strategies are still underdeveloped. In addition, for large and populous countries strong regional and provincial differences were also very much apparent reflecting the local governance systems. Where proactive monitoring was used, a wide variety of responses were recorded with respect to monitoring frequency. A general recommendation of every two weeks is mentioned in the WHO guide, but greater and lesser frequencies were widely reported. Most countries increased the frequency of monitoring seasonally and some implemented a “multi-tier” response system which dynamically modifies the monitoring effort. Cell counts and the use of pigment proxy assessments continue to be the most widely used monitoring methods. Such reliance on what might be called traditional techniques is surprising for planktonic cyanobacteria, given the availability of new genetic tools and remote sensing technologies that are increasingly adopted in research. Clearly, examples of where these new techniques have been integrated into regulatory monitoring frameworks are limited. For benthic cyanobacteria, the reported use of cell counts and toxin analysis is surprising, as neither are particularly effective for assessing risk due to the highly patchy distribution of benthic mats. The fact that taxonomic discrimination during cell count assessments can potentially give some, albeit imperfect, guide to toxigenicity (Ibelings et al. 2015) especially when combined with biomass quantification, may explain, in part, its enduring popularity. Other important factors would be their simplicity and low cost, as well as the inertia to modify regulatory systems. In countries with highly developed and long-established monitoring systems, the deficiencies of a cell and biomass-only approach for risk assessment have become clear (Schurmann et al. 2024). Although they remain irreplaceable as relatively simple, easily deployable methods they are best deployed as a first-tier monitoring response guiding the selective use of more expensive and complicated toxin analysis. The complexity of the relationship between cyanobacteria abundance and toxin concentration and the high likelihood of management over-reaction is a major issue (e.g., Turner et al. 2018) and many respondents were very sensitive to this issue often displaying some frustration about the difficulties of acquiring toxin data. Whilst it is possible that the survey responses in this area may in some cases have involved a misunderstanding with respect to the techniques used by researchers and techniques used in official, or governmental, routine monitoring systems (where they exist) the balance of responses does indicate a limited use of techniques beyond cell counts and biomass estimates globally.

### Which Taxa and Toxins are Reported as Most Problematic and Where?

The results from the survey section on cyanobacterial bloom-forming taxa and associated toxins highlighted some clear patterns; however, these findings should be interpreted with caution. This survey does not constitute an evidence synthesis of peer-reviewed literature. Consequently, reported taxon distributions reflect respondent experience, which is likely shaped by local management issues and emerging concerns rather than true ecological prevalence. A striking example is *Raphidiopsis* (formerly *Cylindrospermopsis*), which is well documented in Australia through extensive bloom records and associated health impacts (Antunes et al. 2015), yet was absent from Australian survey responses. Readers should, therefore, refer to rigorous peer-reviewed syntheses for more definitive assessments of cyanobacterial bloom distributions (e.g. Harke et al. (2016) for *Microcystis*; Kelly et al. (2026) for *Microcoleus*; and Antunes et al. (2015) for *Raphidiopsis*).

Despite these limitations, the most frequently reported planktonic genera (*Microcystis*, *Dolichospermum*, *Planktothrix*, *Aphanizomenon*) and benthic taxa (*Oscillatoria*, *Planktothrix*, *Microcoleus*, *Anabaena*) broadly align with long-recognised bloom-forming taxa, as extensively described in the global literature (Huisman et al. 2018; Wood et al. 2020). The inclusion of *Planktothrix* as a problematic benthic taxon is somewhat unexpected. While *Planktothrix* is known to produce toxins and can form benthic mats (Wood et al. 2010), published reports of this growth form are relatively scarce. Given its biphasic life cycle (Pancrace et al. 2017), with planktonic blooms capable of settling to the benthos, survey responses may reflect this behaviour rather than sustained formation of cohesive benthic mats.

Several cyanobacterial taxa reported including *Pseudanabaena*, *Limnothrix* and *Lyngbya* in planktonic contexts, and *Iningainema* and *Heteroscytonema* in benthic contexts, are not typically considered dominant cyanobacterial bloom-forming taxa. These responses may reflect misinterpretation of the survey question to include background or incidental taxa, or they may indicate localized or emerging bloom events involving atypical taxa that warrant further investigation.

Reported toxin distributions broadly reflect both established cyanotoxin dominance patterns and monitoring bias (Svircev et al. 2019; Wood et al. 2020). Microcystins were the most frequently reported toxins from both planktonic and benthic cyanobacteria, consistent with their global prevalence, structural diversity, and long-standing inclusion in monitoring and regulatory frameworks (Chorus and Welker 2021). Lower reporting of anatoxin-a/homoanatoxin-a, saxitoxins and cylindrospermopsins likely reflects more restricted producer taxa, episodic occurrence and more limited routine surveillance.

### **How do Management Action Thresholds Vary and How are Public Risks Communicated?**

The survey highlighted substantial variation in methods used to trigger management actions and public warning systems for planktonic cyanobacteria, likely reflecting differences in monitoring capacity, regulatory frameworks and local risk perception. Visual detection of surface scums was the most reported trigger, consistent with international guidance emphasising that observable blooms provide an accessible, precautionary indicator of potential risk (United States Environmental Protection Agency 2019; World Health Organization 2021). While visual indicators are widely used, they provide limited quantitative information, and respondents noted that this approach was frequently complemented with measures such as cyanotoxin concentrations, pigment analyses and cell counts or biovolume measurements. These approaches have been applied for decades, often following nationally certified or approved protocols (e.g., Hotzel and Croome 1999). However, they are limited by the small proportion of the waterbody assessed and the requirement for trained personnel. Despite advances in molecular methods and remote sensing, which can overcome some of these limitations by enabling faster, broader-scale monitoring, these technologies were reported by only a few respondents. Adoption of these approaches is expected to increase substantially over the next decade.

Benthic cyanobacteria were monitored less consistently, with action thresholds generally based on mat coverage and toxin concentrations. Compared with planktonic cyanobacteria, benthic blooms are a relatively recent focus (Wood et al. 2020) and the habitats they occupy vary globally (from cobble-bed, fast-flowing rivers in New Zealand to lakes in Germany and Canada; (Kelly et al. 2026) suggest that nuanced, site-specific monitoring approaches are required. Quiblier et al. (2013) highlighted the need for new tools to effectively monitor benthic cyanobacteria, and research and management strategies are likely to advance in this area, leading to a broader range of monitoring approaches. Continued international communication and knowledge sharing will be critical to enable standardisation and comparison of thresholds across regions.

Risk communication strategies are similarly diverse. Social media and static warning signage were the most reported approaches, complemented by educational campaigns, traditional media and online platforms. This aligns with findings from national-scale studies, such as in Canada, where websites, social media, on-site postings and radio broadcasts were identified as the primary channels for communicating risks associated with harmful algal blooms (Rashidi et al. 2021; Hardy et al. 2021). Many of the communication mechanisms mentioned by survey participants are mentioned in D’Anglada (2021), suggesting good uptake of the WHO guidance. Despite the widespread adoption of these strategies, there is currently little empirical evidence evaluating their effectiveness or comparing the relative impact of different methods, highlighting a clear priority for future research.

### Perceptions and Attitudes to Cyanobacteria Risk Management Challenges

Overall, most respondents regarded recreational water management at their location to be inadequate. Africa and South America were the most negative, and Oceania and North America were the most positive. This is a novel observation since no African or South American countries were represented in the survey of recreational water management undertaken by Ibelings et al. (2015). Whilst a relatively low number of reporting countries in two of these four regions, and cultural attitudes, undoubtedly influence the result, there was a clear link to objective assessments of national scientific capacity as indicated by analyses of government spending. International comparisons of R&D spending by country (World bank/OECD, per capita or as % of GDP) document the low spending in the two regions most negative in their perception of management adequacy which will directly relate to the quality of strategies to improve and regulate surface water quality (Aguilera et al. 2023). The serious water challenges and social/economic issues facing many African countries are clearly limiting the development and implementation of effective cyanobacteria risk management (Ndlela et al. 2016). Although projects aiming to share information between African countries, and then internationally, have been attempted in the past (e.g., Codd et al. 2005) the results of this survey indicate that greater effort to improve capacity and access to technical resources especially in Africa is needed (Ndlela et al. 2016).

There was a wide range of interesting and thoughtful responses to the question on how recreational water management could be improved. Suggestions for improvements mostly focused on the desire for more extensive monitoring (i.e., a greater frequency and number of locations) and greater access to cyanotoxin analysis. Approximately half of the countries surveyed reported using cyanotoxin data as a trigger for public health warnings, and the frustration of not always having access to these data was very apparent. Standardized molecular biology protocols have yet to be established for detecting cyanotoxins (Saleem et al. 2023), but may provide a pathway for cheap and accessible, high-throughput testing when sufficiently developed. The current cheapest and most commonly used detection technologies, ELISA-based kit systems and the more sophisticated LC/MS analyses appear to remain out of reach for routine toxin testing, and are therefore best reserved as part of “second tier” analyses in those few countries which have been able to develop local and detailed risk management protocols (See section “What have we learnt about how recreational waters are monitored?”). Our results indicate that such multi-tier responses are very much the minority of global management practise. A dependency of public health authorities on researcher activity and data was pointed out as a management issue in some locations with such dependency regarded as a deficiency due to the short-term and limited project funding researchers typically had access to. The role of non-governmental researchers in risk communication and public education was also highlighted as a deficiency by some respondents, due to the feeling that it indicated an inadequacy in government resource allocation or activity. Such conclusions are of course quite subjective, with many respondents simultaneously highlighting the positive interaction of government regulators and non-governmental researchers in designing and running citizen science programs and the role of researchers in public education programs.

### Conclusions

Our findings highlight widespread variation in monitoring approaches, risk assessment methods and management responses worldwide. This underscores the importance of developing context-specific guidance that is adaptable to local capacities while still aligned with WHO recommendations. Strengthening proactive monitoring, including attention to benthic cyanobacteria, improving access to reliable assessment methods, and standardizing communication strategies could help reduce health risks and improve public confidence in recreational water management. Our results point to the need for targeted assistance for poorer countries in the management of cyanobacteria risks. We hope that this study contributes to the case for increased WHO activity, and other support, in providing technical assistance and capacity building for countries with limited resources, especially where risks may be increasing. Finally, the study emphasizes that expert dissatisfaction with current regimes often stems from perceived increasing risks and insufficient resources. Addressing these gaps will require coordinated international collaboration, knowledge sharing, and sustained investment in both human and technical capacities to ensure safer recreational waters globally.

## Supplementary information


Supplementary information


## Data Availability

An anonymised version of the survey data is available via the Bournemouth University data repository (https://bordar.bournemouth.ac.uk/).
