# Supplementary material for: An International Survey of Cyanobacteria Risk Management in Recreational Waters: Methods, Principal Taxa and Toxins, Challenges and Perspectives
Source: Environ Manage. 2026 Aug 6;76(8):271. doi: 10.1007/s00267-026-02577-z (PMC13447553; doi:10.1007/s00267-026-02577-z)
Supplement: Supplementary file 1 [file 267_2026_2577_MOESM1_ESM.docx]

**Supplementary Material for:**

**An international survey of cyanobacteria risk management in recreational waters: methods, principal taxa and toxins, challenges and perspectives**

Daniel J. Franklin, Laura T. Kelly, Diane M. Orihel,

Jonathan Puddick, Susanna A. Wood

**Supplementary Material S1:** Survey questions and text for the study ‘How are the risks of toxic cyanobacteria in recreational waters being assessed and communicated by countries around the world?’

**Background**

Welcome to our survey on the management of freshwater cyanobacteria in recreational waters. Recreational waters are defined by the World Health Organization (WHO) as those used for activities that make people happy and healthy such as swimming, fishing, other water-based activities and sports. We are a team of researchers interested in what we can learn from each other in the management of the risks associated with this potentially serious issue.

We estimate the survey will take about 10 minutes to complete. There is no commitment to take part in this survey. All responses are optional, except for your country, as we are interested in how cyanobacteria management varies across the world. If you cannot answer a question please simply leave it blank. If you give your name and email address within the survey a member of the project team may contact you for further discussions. We will store all data securely and will not retain data inappropriately. Summarised and anonymised results may be included in public reporting. Our aim is to report the survey results in the literature and create a freely available risk communication resource for the benefit of all.

Thank you.

Dan Franklin (Bournemouth University, United Kingdom), Susie Wood (Lincoln University, Aotearoa-New Zealand), Laura Kelly and Jonathan Puddick (Cawthron Institute, Aotearoa-New Zealand) and Diane Orihel (Queen’s University, Canada).

**Consent**

1. I understand the [participant information](https://docs.google.com/document/d/1Pb14fQj70PGgN_nR0CvsnyJz1UgSM4ri/edit?usp=drive_link&ouid=110201077936072438108&rtpof=true&sd=true) and that my data may be included in an anonymised form within a dataset to be archived at an online research data repository at Bournemouth University.

**About you**

2. Full Name (Optional)

3. Email Address (Optional)

4. Which country are you reporting on?

5. We understand there can be significant variation within countries as well as between countries. If you are reporting from a large or populous country, please give the region you are reporting from (e.g. Chinese province, U.K. nation, U.S. state). In this survey we will henceforth understand this to be “your location”

6. How confident are you in describing how cyanobacteria risks are assessed and communicated? (Very/Somewhat/Not confident)

**About cyanobacteria in recreational waters at your location**

7. Is there routine monitoring for cyanobacteria in recreational waters at your location? That is, monitoring undertaken prior to the development of an obvious (visual) cyanobacteria bloom? (Yes/No/Unsure)

8. Are both benthic and planktonic cyanobacteria monitored? (Yes, both are assessed/No, only planktonic types are assessed/Unsure)

Benthic = growing attached to a substrate (e.g. sand, rocks, plants) in the river or lake. Planktonic = growing in the water or floating. Sometimes, this type accumulates at the water's edge as a “scum” or “crust”.

9. Are both river and lake sites monitored? (Lakes only/Lakes and rivers/Unsure)

10. **Planktonic** - What methods are used to monitor cyanobacteria in recreational waters at your location, tick all that apply. (Chlorophyll/phycocyanin measurements/Turbidity measurements/Samples for cell counts/Samples for cell volumes/Samples for cyanotoxin analysis/Samples for the molecular detection of toxigenicity/Continuous monitoring via sensor buoys/Satellite imagery/Drone imagery/Visual surveys/Others)

11. **Benthic** - What methods are used to monitor cyanobacteria in recreational waters at your location, tick all that apply. (Chlorophyll/phycocyanin measurements/Samples for cell counts/Samples for cyanotoxins/Samples for molecular analysis/ Drone imagery/Visual surveys/Others)

12. How frequently is monitoring for cyanobacteria carried out at your location? Example: monthly during the summer.

13. Please select the **planktonic** cyanobacteria that form blooms or cause problems at your location. Please tick all that apply. (Aphanizomenon/Chrysosporum/Cuspidothrix/Dolichospermum/Gloeeotrichia/Microcystis/Nodularia/Planktothrix/Raphidiopsis/Sphaerospermopsis/Synechococcus/Trichodesmium/Woronichinia/Other (please specify below))

14. Additional comments if Other was selected.

15. Please select the **benthic** cyanobacteria that form blooms or cause problems at your location. Please tick all that apply. (Anabaena/Microcoleus/Phormidium/Microseira (Lyngbya)/Nostoc/Oscillatoria/Planktothrix/Scytonema/Tychonema/Wollea/ Other (please specify below))

16. Additional comments if Other was selected.

17. Please select the cyanotoxin(s) from **planktonic** cyanobacteria that are detected at your location. (Aetokthonotoxin/Anatoxin-a/Homoanatoxin-a/Aplysiatoxins/Beta-methylamino-L-alanine (BMAA)/Cylindrospermopsins/Guanitoxin (anatoxin-a(S))/Lyngbyatoxins/Microcystins/Nodularins/Saxitoxins/Other)

18. Additional comments if Other was selected.

19. Please select the cyanotoxin(s) from **benthic** cyanobacteria that are detected at your location. (Aetokthonotoxin/ Anatoxin-a/Homoanatoxin-a/Aplysiatoxins/ Beta-methylamino-L-alanine (BMAA)/ Cylindrospermopsins/ Guanitoxin (anatoxin-a(S))/ Lyngbyatoxins/Microcystins/ Nodularins/ Saxitoxins/ Other)

20. Additional comments if Other was selected.

**Cyanobacteria management during blooms/periods of elevated concern at your location**

21. **Planktonic** - What data (if any) triggers action, in the sense of public warnings, at your location? Tick all that apply. (Presence of scums/Cyanobacteria biovolumes/Cyanobacteria cell counts/Chlorophyll/phycocyanin measurements/Molecular measurements of taxonomy/toxigenicity/Cyanotoxin concentrations/App-based citizen science measurements/reports/Other (please specify below)/None – no public warning system operates)

22. Additional comments if any.

23. **Benthic** - What data (if any) triggers action, in the sense of public warnings, at your location? Tick all that apply. (% benthic mat cover/Cyanotoxin concentration/Molecular measurements/Other (please specify below)/ None – no public warning system operates)

24. Additional comments if any.

25. If there is a trigger data value (e.g. >20,000 cells/ml), or other criterion (e.g. presence of shoreline scums), for public warnings at your location please give further brief details. If possible, please give links to any public documents detailing the warning strategy at your location.

26. How are the risks of cyanobacteria communicated to the public at your location? Tick all that apply. (Static signs (permanent)/Static signs (permanent/modifiable)/Static signs (temporary)/Social media alerts/ Educational/public awareness campaigns/Other (please specify below))

27. Additional comments if any.

28. Asides from public warnings, are any other management interventions used at your location to reduce risk? E.g. waterbody closures.

Please specify.

**Your opinion on how cyanobacteria management and risk communication could be improved at your location**

29. Do you consider the management of the risks to recreational water users at your location to be adequate? (Yes/No/Unsure)

30. Additional comments if any.

31. Would you like to see any changes to the management of cyanobacteria risks in recreational waters at your location? (Yes/No/Unsure)

32. Additional comments if any.

**Final comments and information sharing**

33. If you would like to add anything to your responses, or if you have any comments or suggestions for the project team, please elaborate below.

Many thanks for taking the time to complete this survey!

Click the submit button below submit your answers.

**Supplementary Material S2:** Summary of responses to Question 12 relating to how frequently monitoring is undertaken for benthic and planktonic cyanobacteria at the location where the respondent was based.

| **Country** | **Spring / Summer / Autumn** | | | | | | | **Winter** | | | | **Year Round (samples per year)** | | | | | |
| --- | --- | --- | --- | --- | --- | --- | --- | --- | --- | --- | --- | --- | --- | --- | --- | --- | --- |
|  | **Daily** | **2 per Week** | **Weekly** | **Fortnightly** | **Monthly** | **Responsive** | **None** | **Weekly** | **Monthly** | **3-Monthly** | **None** | **1** | **3** | **4** | **8** | **12** | **52** |
| Argentina |  |  |  | 1 |  | 1 |  |  | 1 |  |  |  |  |  |  |  |  |
| Australia |  |  | 1 |  | 1 | 1 |  |  |  |  |  |  |  |  |  |  |  |
| Austria |  |  |  | 1 |  |  |  |  |  |  |  |  |  |  |  |  |  |
| Belgium |  |  | 2 | 1 |  | 1 |  |  |  |  |  |  |  |  |  |  |  |
| Brazil |  |  | 1 |  | 3 | 1 |  |  |  |  |  |  |  |  |  |  |  |
| Canada | 2 | 2 | 3 | 2 | 4 | 8 |  |  | 1 |  |  |  |  |  |  |  |  |
| China |  |  |  |  | 1 |  |  |  |  |  |  |  |  |  |  | 1 |  |
| Cyprus |  |  |  |  |  | 1 |  |  |  |  |  |  |  | 1 |  |  |  |
| Denmark |  |  |  |  |  | 1 |  |  |  |  |  |  |  |  |  |  |  |
| Ecuador |  |  |  |  |  | 1 |  |  |  |  |  |  |  |  |  |  |  |
| Germany |  |  |  | 2 | 1 |  |  |  |  |  |  |  |  |  |  |  |  |
| Ghana |  |  |  |  |  | 1 |  |  |  |  |  |  |  |  |  |  |  |
| Guatemala |  |  |  |  | 1 |  |  |  |  |  |  |  |  |  |  |  |  |
| Finland |  |  | 1 |  | 1 |  |  |  |  |  |  |  |  |  |  |  |  |
| France |  |  | 1 | 2 | 3 |  |  |  |  |  | 1 |  |  |  |  |  |  |
| Israel |  |  |  |  |  |  |  |  |  |  |  |  |  |  |  |  | 1 |
| India |  |  |  |  |  |  |  |  |  |  |  | 1 |  |  |  |  |  |
| Italy |  |  |  | 1 | 1 |  |  |  | 1 |  |  |  |  |  |  |  |  |
| Japan |  |  | 1 | 2 | 2 |  |  |  |  |  |  |  | 1 |  |  |  |  |
| Kenya |  |  |  |  |  | 1 |  |  |  |  |  |  |  | 2 |  |  |  |
| Macedonia |  |  |  |  |  |  | 1 |  |  |  |  |  |  |  |  |  |  |
| Malaysia |  |  |  |  |  |  |  |  |  |  |  | 1 |  |  |  |  |  |
| Namibia |  |  | 1 |  | 1 |  |  |  |  |  |  |  |  |  |  |  |  |
| Netherlands |  | 1 | 1 | 1 | 1 |  |  |  |  |  |  |  |  |  |  |  |  |
| New Zealand |  |  | 2 | 1 | 1 | 1 |  |  | 1 |  |  |  |  |  |  |  |  |
| Norway |  |  | 1 |  | 1 |  |  |  |  |  | 1 |  |  |  |  |  |  |
| Paraguay |  |  | 1 |  |  | 1 |  |  |  | 1 |  |  |  |  |  |  |  |
| Poland |  |  |  | 1 | 2 |  |  |  |  |  |  |  |  |  |  |  |  |
| Portugal |  |  |  |  | 1 |  |  |  |  |  |  |  |  |  |  |  |  |
| Romania |  |  |  |  |  |  | 1 |  |  |  |  |  |  |  |  |  |  |
| Russia |  |  |  |  |  |  |  |  |  |  |  |  |  |  |  |  | 1 |
| Singapore |  |  |  |  |  |  |  |  |  |  |  |  |  |  |  | 1 |  |
| South Africa |  |  |  |  |  |  | 1 |  |  |  |  |  |  | 1 |  | 1 |  |
| South Korea |  |  |  |  |  |  |  |  |  |  |  |  |  |  |  |  |  |
| Spain |  |  |  |  | 2 | 1 |  |  |  |  |  |  |  | 1 |  |  |  |
| Sweeden |  |  | 1 | 1 |  |  |  |  |  |  |  |  |  |  |  |  |  |
| Switzerland |  |  |  |  |  |  |  |  |  |  |  |  |  |  | 1 |  |  |
| Ukraine |  |  |  |  |  |  | 1 |  |  |  | 1 |  |  |  |  |  |  |
| United Kingdom |  |  |  |  | 4 | 5 |  |  |  |  |  |  |  |  |  |  |  |
| USA |  |  | 9 | 5 | 9 | 10 |  |  | 2 |  |  | 1 |  | 1 | 1 | 1 |  |
| Uruguay |  | 1 | 2 |  |  |  |  | 1 | 1 |  |  |  |  |  |  |  |  |
| **Total Countries** | **1** | **3** | **15** | **13** | **19** | **15** | **5** | **1** | **6** | **1** | **3** | **3** | **1** | **6** | **2** | **4** | **2** |

**Supplementary Material S3:** Countries for which anatoxin-a or homoanatoxin-a were identified by respondents as posing a risk for planktonic (top) and benthic (bottom) cyanobacteria.

**Supplementary Material S4:** Countries for which beta-methylamino-l-alanine (BMAA) were identified by respondents as posing a risk for planktonic (top) and benthic (bottom) cyanobacteria.

**Supplementary Material S5:** Countries for which cylindrospermopsins were identified by respondents as posing a risk for planktonic (top) and benthic (bottom) cyanobacteria.

**Supplementary Material S6:** Countries for which nodularins were identified by respondents as posing a risk for planktonic (top) and benthic (bottom) cyanobacteria.

**Supplementary Material S7:** Countries for which saxitoxins were identified by respondents as posing a risk for planktonic (top) and benthic (bottom) cyanobacteria.

**Supplementary Material S8:** Summary of responses to Questions 21 and 22 relating to triggers for action (in the sense of public warnings) for planktonic cyanobacteria.

| **Country** | **Presence of scums / visual presence of blooms** | **Cyanobacteria biovolumes** | **Cyanobacteria cell counts** | **Chlorophyll / phycocyanin measurements** | **Molecular measurements of taxonomy / toxigenicity** | **Cyanotoxin concentrations** | **App-based citizen science measurements / reports** | **Reports from the public** | **Reports of human illness and / or animal deaths** | **Secchi disc measurements** | **Satellite imagery** | **On-site rapid tests** | **Bad odours** | **Planktonic cyanobacteria not assessed** | **None – No public warning system** |
| --- | --- | --- | --- | --- | --- | --- | --- | --- | --- | --- | --- | --- | --- | --- | --- |
| Argentina | 2 | 1 | 2 |  |  | 2 |  |  |  |  | 1 |  |  |  |  |
| Australia | 1 |  | 1 |  |  | 1 | 1 |  |  |  |  |  |  |  |  |
| Austria | 1 |  |  | 1 |  | 1 |  |  |  |  |  |  |  |  |  |
| Belgium | 2 |  |  | 1 |  | 3 | 1 |  |  |  |  |  |  |  |  |
| Brazil | 3 | 2 | 2 | 1 |  | 3 |  |  |  |  |  |  |  |  |  |
| Canada | 14 | 2 | 7 | 3 | 1 | 10 | 1 | 3 |  |  |  | 1 |  |  | 1 |
| Chile | 1 |  | 1 |  |  | 1 |  |  |  |  |  |  |  |  |  |
| China | 1 |  |  |  |  | 1 |  |  |  |  |  |  |  |  | 1 |
| Croatia | 1 | 1 | 1 | 1 |  |  |  |  |  |  |  |  |  |  |  |
| Cyprus | 1 | 1 | 1 | 1 |  | 1 |  |  |  |  |  |  |  |  |  |
| Denmark | 1 |  | 1 |  |  |  |  |  |  |  |  |  |  |  |  |
| Ecuador |  |  |  |  |  |  |  |  |  |  |  |  |  |  | 1 |
| Estonia | 1 | 1 |  | 1 |  |  |  |  |  |  |  |  |  |  |  |
| Finland | 2 | 1 |  | 1 |  |  |  |  |  |  |  |  |  |  |  |
| France | 5 | 6 | 1 | 4 |  | 6 |  |  |  |  |  |  |  |  |  |
| Germany | 4 | 1 | 1 | 2 |  | 2 |  |  |  | 1 |  |  |  |  |  |
| Ghana | 1 |  |  |  |  |  |  |  |  |  |  |  |  |  |  |
| Greece | 1 |  |  | 1 |  | 1 |  |  |  |  |  |  |  |  |  |
| Guatemala |  |  | 1 | 1 |  |  |  |  |  |  |  |  |  |  |  |
| Iran |  |  | 1 | 1 |  |  |  |  |  |  |  |  |  |  |  |
| Israel | 1 | 1 | 1 | 1 |  | 1 |  |  |  |  |  |  |  |  |  |
| India |  |  |  |  |  |  |  |  |  |  |  |  |  |  |  |
| Italy | 2 |  | 2 | 1 |  | 1 |  |  |  |  |  |  |  |  |  |
| Japan | 6 |  |  | 1 |  |  |  |  |  |  |  |  | 1 |  | 1 |
| Kenya | 2 | 1 |  | 1 |  | 2 |  |  |  |  |  |  |  |  |  |
| Macedonia |  |  |  |  |  |  |  |  |  |  |  |  |  |  |  |
| Malaysia |  |  |  |  |  | 1 |  |  |  |  |  |  |  |  |  |
| Namibia |  |  | 1 | 1 |  |  |  |  |  |  |  |  |  |  |  |
| Netherlands | 3 | 3 | 2 | 3 |  | 1 |  | 1 |  |  |  |  |  |  |  |
| New Zealand | 2 | 2 | 1 | 1 |  |  |  |  |  |  |  |  |  |  | 2 |
| Norway | 2 | 2 |  |  |  | 2 | 2 |  |  |  |  |  |  |  |  |
| Paraguay |  |  | 1 |  |  | 1 |  |  |  |  |  |  |  | 1 |  |
| Poland | 2 | 1 |  |  |  |  |  |  |  |  |  |  |  |  |  |
| Portugal | 1 |  | 1 | 1 |  | 1 |  |  |  |  |  |  |  |  |  |
| Romania | 1 |  | 1 | 1 |  | 1 |  |  |  |  |  |  |  |  |  |
| Russia |  |  |  |  |  |  | 1 |  | 1 |  |  |  |  |  | 2 |
| Singapore | 1 |  | 1 |  |  |  |  |  |  |  |  |  |  |  |  |
| South Africa |  |  | 1 | 1 |  | 1 |  |  |  |  |  |  |  |  |  |
| South Korea |  |  | 1 |  |  | 1 |  |  |  |  |  |  |  |  |  |
| Spain | 4 |  | 1 | 2 |  | 1 |  |  |  |  |  |  |  |  |  |
| Sweeden | 2 |  |  |  |  |  |  |  |  |  |  |  |  |  |  |
| Switzerland | 1 |  |  |  |  |  |  |  |  |  |  |  |  |  |  |
| Thailand | 1 |  | 1 | 1 |  | 1 |  |  |  |  |  |  |  |  |  |
| Ukraine |  | 1 | 1 | 1 |  |  |  |  |  |  |  |  |  |  | 1 |
| United Kingdom | 9 | 4 | 6 | 1 |  | 1 | 3 | 1 |  |  |  |  |  |  | 1 |
| USA | 24 | 3 | 12 | 8 | 2 | 25 | 5 |  | 3 | 1 | 2 | 1 |  |  |  |
| Uruguay | 3 |  |  | 1 |  | 1 |  |  |  |  |  |  |  |  |  |
| **Total Countries** | **35** | **18** | **28** | **28** | **2** | **28** | **7** | **3** | **2** | **2** | **2** | **2** | **1** | **1** | **8** |

**Supplementary Material S9:** Summary of responses to Questions 23 and 24 relating to triggers for action (in the sense of public warnings) for benthic cyanobacteria.

| **Country** | **%benthic mat cover** | **Cyanotoxin concentrations** | **Molecular measurements** | **Reports of human illness and / or animal deaths** | **Detached mats** | **Taxonomic screening by microscopy** | **Reports from the public** | **None – No public warning system** |
| --- | --- | --- | --- | --- | --- | --- | --- | --- |
| Canada | 2 | 2 | 1 |  |  | 1 | 2 | 2 |
| France | 2 | 2 |  | 2 |  |  |  |  |
| Ghana |  |  |  |  |  |  |  | 1 |
| Iran | 1 |  |  |  |  |  |  |  |
| Japan |  |  |  |  |  |  |  | 1 |
| Kenya |  | 1 |  |  |  |  |  |  |
| Namibia |  | 1 |  |  |  |  |  |  |
| Netherlands | 2 |  |  |  |  |  |  |  |
| New Zealand | 4 | 1 |  |  | 1 |  |  |  |
| Norway |  |  |  |  |  |  |  | 1 |
| Russia |  |  |  |  |  |  |  | 1 |
| Ukraine |  |  |  |  |  |  |  | 1 |
| United Kingdom | 2 | 2 |  |  |  |  |  |  |
| USA | 7 | 10 |  | 1 | 1 |  |  | 1 |
| **Total Countries** | **7** | **7** | **1** | **2** | **2** | **1** | **1** | **7** |

**Supplementary Material S10:** Summary of responses to Questions 26 and 27 related to risk communication mechanisms adopted at the location of the respondent

| **Country** | **Static signs (permanent)** | **Static signs (permanent / modifiable)** | **Static signs (temporary)** | **Social media alerts** | **Educational / public awareness campaigns** | **Websites** | **Interactive maps** | **Specialised Communication / Reporting Apps** | **Traditional media (television, radio, newspaper)** | **Fliers / pamphlets / email send-outs** | **Direct communication with high-risk groups** | **Technical notes / research briefs** | **Don’t know** | **No risk communication undertaken** |
| --- | --- | --- | --- | --- | --- | --- | --- | --- | --- | --- | --- | --- | --- | --- |
| Argentina |  |  | 1 | 1 | 1 |  |  |  |  |  |  |  |  |  |
| Australia | 1 |  | 2 | 1 |  |  |  |  | 1 |  |  |  |  |  |
| Austria |  |  | 1 |  |  |  |  |  |  |  |  |  |  |  |
| Belgium | 2 | 1 | 2 | 3 | 1 | 1 |  |  | 1 | 1 |  |  |  |  |
| Brazil | 1 | 1 |  |  | 2 |  |  |  |  |  |  | 2 |  |  |
| Canada | 8 | 4 | 12 | 16 | 11 | 6 | 1 |  | 1 |  |  |  | 1 |  |
| Chile |  | 1 |  | 1 |  |  |  |  |  |  |  |  |  |  |
| China |  |  | 1 | 1 | 1 |  |  |  |  |  |  |  |  |  |
| Cyprus | 1 | 1 |  |  |  |  |  |  |  |  |  |  |  |  |
| Denmark |  | 1 | 1 | 1 |  |  |  |  |  |  |  |  |  |  |
| Ecuador |  |  |  |  |  |  |  |  | 1 |  |  |  |  |  |
| Estonia |  |  | 1 | 1 |  |  |  |  |  |  |  |  |  |  |
| Finland |  | 1 | 2 | 1 | 1 |  |  |  |  |  |  |  |  |  |
| France |  | 1 | 4 | 4 | 1 | 1 |  |  |  |  |  |  |  |  |
| Germany | 1 | 2 | 3 | 2 | 1 | 1 |  |  |  |  |  |  |  |  |
| Guatemala |  |  |  | 1 |  |  |  |  |  |  |  |  |  |  |
| India |  | 1 |  |  |  |  |  |  |  |  |  |  |  |  |
| Iran |  |  | 1 |  |  |  |  |  |  |  |  |  |  |  |
| Israel |  |  | 1 | 1 |  |  |  |  |  |  |  |  |  |  |
| Italy |  |  | 1 | 1 |  |  |  |  |  |  |  |  |  |  |
| Japan |  |  |  | 4 | 1 |  |  |  | 1 |  |  |  |  | 2 |
| Kenya |  |  |  | 1 | 3 |  |  |  |  |  |  |  |  |  |
| Macedonia |  |  |  |  |  |  |  |  |  |  |  |  |  | 1 |
| Malaysia |  |  |  |  | 1 |  |  |  |  |  |  |  |  |  |
| Namibia | 1 |  |  | 1 |  |  |  |  |  |  |  |  |  |  |
| Netherlands | 2 | 1 | 2 | 1 | 1 | 3 |  |  |  |  |  |  |  |  |
| New Zealand | 4 | 2 | 3 | 4 | 4 |  | 1 |  | 1 |  | 2 |  |  |  |
| Norway | 1 | 1 | 2 | 2 |  | 1 |  | 1 | 1 |  |  |  |  |  |
| Paraguay |  |  |  | 1 | 1 | 1 |  |  |  |  |  |  |  |  |
| Poland |  |  | 1 | 2 | 1 |  |  |  |  |  |  |  |  |  |
| Portugal |  | 1 | 1 |  |  |  |  |  |  |  |  |  |  |  |
| Romania |  |  |  | 1 |  |  |  |  |  |  |  |  |  |  |
| Russia | 1 |  |  | 1 |  |  |  |  |  |  |  |  |  |  |
| Singapore |  |  |  |  |  |  |  |  |  |  |  |  |  | 1 |
| Slovenia |  |  |  | 1 | 1 |  | 1 |  |  | 1 | 1 |  |  |  |
| South Africa |  |  |  |  |  | 1 |  |  |  |  |  |  |  | 1 |
| South Korea |  |  |  | 1 | 1 |  |  |  |  |  |  |  |  |  |
| Spain | 1 | 1 | 1 | 1 |  | 1 |  |  | 1 |  |  |  |  |  |
| Sweeden |  |  | 1 | 1 |  | 2 |  |  |  |  |  |  |  |  |
| Switzerland |  |  | 2 | 1 | 1 |  |  |  | 2 |  |  |  |  |  |
| Thailand |  |  |  | 1 |  |  |  |  |  |  |  |  |  |  |
| Ukraine |  |  |  | 1 |  |  |  |  |  |  |  |  |  |  |
| United Kingdom | 3 | 5 | 9 | 5 | 1 |  |  |  |  |  | 1 |  |  |  |
| USA | 18 | 7 | 25 | 25 | 19 | 7 | 3 |  | 6 | 2 |  |  |  |  |
| Uruguay |  | 1 | 3 |  | 1 | 2 |  | 1 |  |  |  |  |  |  |
| **Total Countries** | **14** | **18** | **25** | **33** | **21** | **12** | **4** | **2** | **10** | **3** | **3** | **1** | **1** | **4** |

**Supplementary Material S11** – Weblinks to cyanobacteria management documents supplied by survey respondents (note: documents that were not publicly accessible were omitted).

**Argentina**

Cyanobacteria alerts website (in Spanish) – <https://www.gba.gob.ar/cianobacterias>

Cyanobacteria information website (in Spanish) – <https://caru.org.uy/nuevositio/2025/02/04/prevencion-ante-la-presencia-de-cianobacterias-en-el-rio/>

**Austria**

Cyanobacteria information sheet (in German) – <https://www.ages.at/download/sdl-eyJ0eXAiOiJKV1QiLCJhbGciOiJIUzI1NiJ9.eyJpYXQiOjE2MDk0NTkyMDAsImV4cCI6NDA3MDkwODgwMCwidXNlciI6MCwiZ3JvdXBzIjpbMCwtMV0sImZpbGUiOiJmaWxlYWRtaW4vQUdFU18yMDIyLzJfTUVOU0NIL0tyYW5raGVpdC9cdTAwZDZmZmVudGxpY2hlX0dlc3VuZGhlaXRfU2VydmljZXMvQmVyaWNodGVfRm9sZGVyLzA3X1Vtd2VsdF9XYXNzZXJfQ3lhbm9iYWt0ZXJpZW5fQkYucGRmIiwicGFnZSI6OTEyfQ.G3KPgMP0BI4n3nZibt0V7iDq-93GxPSy_e_7IbpfY28/07_Umwelt_Wasser_Cyanobakterien_BF.pdf>

Bathing waters database for Austria (in German) – <https://www.ages.at/umwelt/wasser/badegewaesser-monitoring>

**Belgium**

Water quality information website – <https://www.issep.be/en/water-quality/>

**Canada**

Information on blue-green algae / cyanobacteria and cyanobacteria monitoring data for Nova Scotia – <https://novascotia.ca/blue-green-algae/>

Information on the blue-green algae / cyanobacteria monitoring program for Halifax – <https://www.halifaxwater.ca/blue-green-algae>

Information on the blue-green algae / cyanobacteria from Ontario – <https://www.ontario.ca/page/blue-green-algae#section-3>

Cyanobacteria identification guide (in French) – <https://www.environnement.gouv.qc.ca/eau/eco_aqua/cyanobacteries/guide-identif.pdf>

Information on the blue-green algae / cyanobacteria from Québec – <https://www.environnement.gouv.qc.ca/eau/algues-bv/index.asp>

Cyanobacteria / blue-green algae health advisories for Alberta Health Services – <https://www.albertahealthservices.ca/news/bga.aspx>

Active health advisories for Alberta Health Services – <https://www.albertahealthservices.ca/news/Page1926.aspx>

Interactive map communicating water quality information for public beaches in the Saskatchewan region – <https://gis.saskatchewan.ca/portal/apps/instant/sidebar/index.html?appid=47584e5a0a7d48ada28eb6e58ea096c5>

Information on the healthy beaches program for the Saskatchewan region – <https://www.saskatchewan.ca/residents/environment-public-health-and-safety/environmental-health/healthy-beach-program>

Data on cyanobacterial blooms in Alberta recreational waters – <https://open.alberta.ca/opendata/cyanobacterial-blooms-in-alberta-recreational-waters>

Guidelines for Canadian recreational water quality: Summary document – <https://www.canada.ca/en/health-canada/services/publications/healthy-living/guidelines-canadian-recreational-water-quality-summary-document.html>

Information on the healthy beaches program for the Saskatchewan region – <https://www.saskatchewan.ca/residents/environment-public-health-and-safety/environmental-health/healthy-beach-program>

**Cyprus**

News article; “Mass fish die-off at Athalassa Lake under investigation” – <https://cyprus-mail.com/2024/10/18/mass-fish-die-off-at-athalassa-lake-under-investigation>

News article; “Athalassa national park in troubled waters” – <https://knews.kathimerini.com.cy/en/news/athalassa-national-park-in-troubled-waters>

News article; “Polluted Polemidia must be off limits” – <https://archive.cyprus-mail.com/2017/03/09/polluted-polemidia-must-off-limits/>

Archive of presentations on water quality – <https://www.moa.gov.cy/moa/wdd/wdd.nsf/All/56BFD839913D6065C2258456002A3114?OpenDocument>

**Finland**

Bathing water profile for Pikkukoski Beach, Helsinki (in Finnish) – <https://www.hel.fi/static/ymk/uimavedet/uimavesiprofiili-pikkukoski.pdf>

Example of swimming water quality report – <https://www.hel.fi/static/liitteet/kaupunkiymparisto/kulttuuri-ja-vapaa-aika/uimarannat/Uimavedenlaatu_2024.pdf>

**France**

Technical report; “Risk assessment related to cyanobacteria and their toxins in freshwater” (in French) – <https://www.anses.fr/fr/system/files/EAUX2016SA0165Ra.pdf>

Information website for the association for the management of bathing areas in the Landes region (in French) – <https://www.smgbl.fr/Baigneur>

**Germany**

Information on bathing water monitoring from the State Office for Health and Social Affairs (in German) – <https://www.berlin.de/lageso/gesundheit/gesundheitsschutz/badegewaesser/badegewaesserueberwachung/>

Article; “Recommendation for the protection of bathers from cyanobacteria and cyanobacterial toxins” (in German) – <https://link.springer.com/article/10.1007/s00103-024-03944-z>

**Greece**

Scientific article; “Cyanobacterial toxins and peptides in Lake Vegoritis, Greece” – <https://doi.org/10.3390/toxins13060394>

**Italy**

Article; “Cyanobacteria blooms in water: Italian guidelines to assess and manage the risk associated to bathing and recreational activities” – <http://dx.doi.org/10.1016/j.scitotenv.2017.03.232>

**Netherlands**

“Cyanobacteria Protocol 2020” from the National Institute for Public Health and the Environment – <https://www.rivm.nl/bibliotheek/rapporten/2020-0167.pdf>

Information on the blue-green algae platform (in Dutch) – <https://www.stowa.nl/onderwerpen/waterkwaliteit/van-kennis-naar-praktijk/platform-blauwalgen>

Interactive map presenting information on swimming spots in the Netherlands– <https://www.zwemwater.nl/>

**New Zealand**

Aotearoa New Zealand Guidelines for Cyanobacteria in Recreational Freshwaters – <https://environment.govt.nz/assets/publications/Cyanobacteria-Guidelines_ME1851.pdf>

**Paraguay**

Scientific article; “Cyanobacterial bloom monitoring and assessment in Latin America” – <https://doi.org/10.1016/j.hal.2023.102429>

**Poland**

Bathing information from the Chief Sanitary Inspectorate (in Polish) – <https://sk.gis.gov.pl/>

**Romania**

News article; “Alert on the coast: Toxic algae found in the Black Sea” (in Romanian) – <https://www.google.com/amp/s/www.gandul.ro/actualitate/alerta-pe-litoral-au-fost-gasite-alge-toxice-20225345/amp>

News article; “ALERT! Toxic algae in the Black Sea. Poisons are reaching Romanians' plates” (in Romanian) – <https://www.romaniatv.net/alerta-alge-toxice-in-marea-neagra-otravurile-ajung-in-farfuriile-romanilor_396332.html?uord=Oqa4lFGnhIsoIB%3D3ZEtiPWRPT%3DJQUPuZ9cApPblvjVh0tlwlJ6CtM22LWImlPUFTQ1y-XdG/PcRyp6OXmBbbIpGtNscz7JGEHEJQI1FrPX84OWGliYB4g_vvTFLlYUkA9mJQIUGF>

**Ukraine**

Scientific article; “Interactions between aquatic plants and cyanobacterial blooms in freshwater reservoir ecosystems” – <https://www.mdpi.com/2073-4441/15/4/672>

**United Kingdom (UK)**

Public health guidance for cyanobacteria from Public Health Scotland (Version 1) – <https://publichealthscotland.scot/publications/public-health-guidance-for-cyanobacteria/public-health-guidance-for-cyanobacteria/>

“Guidelines on recreational water quality: Volume 1 Coastal and fresh waters” from the World Health Organization – <https://www.who.int/publications/i/item/9789240031302>

Guidance on “Algal blooms: Advice for the public and landowners” from the UK Environment Agency – <https://www.gov.uk/government/publications/algal-blooms-advice-for-the-public-and-landowners/algal-blooms-advice-for-the-public-and-landowners>

Information website on cyanobacteria from the UK Environment Agency – <https://engageenvironmentagency.uk.engagementhq.com/blue-green-algae-in-cumbria-and-lancashire>

Information on the “Bloomin’ Algae” app from the UK Centre for Ecology and Hydrology – <https://www.ceh.ac.uk/our-science/projects/bloomin-algae>

**United States of America (USA)**

Algal bloom dashboard from the Florida Department of Environmental Protection – <https://floridadep.gov/AlgalBloom>

Harmful algal blooms toolkit from the Washington State Department of Health – <https://doh.wa.gov/community-and-environment/contaminants/cyanobacteria/resources>

Information on planktonic cyanobacteria from the State of California Water Quality Monitoring Council – <https://mywaterquality.ca.gov/habs/resources/response-guidance.html#planktonic>

Information on benthic cyanobacteria from the State of California Water Quality Monitoring Council – <https://mywaterquality.ca.gov/habs/resources/response-guidance.html#benthic-cyanobacteria>

Harmful algal blooms response guidance from the State of California Water Quality Monitoring Council – <https://mywaterquality.ca.gov/habs/resources/response-guidance.html>

Advisory guidelines for the Oregon cyanobacteria harmful algae bloom surveillance program – <https://www.oregon.gov/oha/PH/HEALTHYENVIRONMENTS/RECREATION/HARMFULALGAEBLOOMS/Documents/Advisory%20Guidelines%20for%20Harmful%20Cyanobacteria%20Blooms%20in%20Recreational%20Waters.2024_Final.pdf>

Cyanobacteria advisories from the Oregon Health Authority – <https://www.oregon.gov/oha/ph/healthyenvironments/recreation/harmfulalgaeblooms/pages/blue-greenalgaeadvisories.aspx>

Interactive map of water quality status for harmful algal blooms and beach conditions in Florida – <https://www.floridahealth.gov/environmental-health/aquatic-toxins/where-are-habs.html>

Nevada cyanobacterial harmful algal bloom strategic response plan (May 2024) – <https://ndep.nv.gov/uploads/water-rsl-hab-docs/NV_HAB_SRP_Package_(Final_2024).pdf>

Blue-green algae information website from the Wisconsin Department of Natural Resources – <https://dnr.wisconsin.gov/topic/lakes/bluegreenalgae>

Cyanobacterial harmful algal blooms information website from the Wisconsin Department of Health Services – <https://www.dhs.wisconsin.gov/algae/index.htm>

State and tribal toxin thresholds and harmful algal bloom advisory decision methods for USA – <https://www.epa.gov/habs/state-tribal-toxin-thresholds-and-assessment-methods>

Harmful algal bloom reports map – <https://mywaterquality.ca.gov/habs/resources/reports-map/>

California satellite harmful algal bloom map – <https://mywaterquality.ca.gov/habs/resources/satellite-map.html>

Weekly updates for the freshwater harmful algal bloom program – <https://docs.google.com/spreadsheets/d/1fH1E6R1_pqjYIyiytEuARsLGD2EOjtul2NJtkdw-QxY/edit?usp=sharing>

Current public health advisories for the Clark County (Washington State, USA) – <https://clark.wa.gov/public-health/public-beaches>

Public health advisory protocol and dashboard for the Clark County (Washington State, USA) – <https://clark.wa.gov/public-health/advisory-protocol>

Interactive map of harmful algal blooms in Nevada – <https://app.powerbigov.us/view?r=eyJrIjoiOGEzY2FkMDAtMzNhNC00OTYyLTg0ZDYtYjc2YThkNWI4NWZjIiwidCI6ImU0YTM0MGU2LWI4OWUtNGU2OC04ZWFhLTE1NDRkMjcwMzk4MCJ9>

Harmful algal blooms dashboard for Navada Division of Environmental Protection – <https://ndep.nv.gov/water/rivers-streams-lakes/water-quality-monitoring/harmful-algal-bloom-program#Current%20Advisories%20(Spring%20through%20Summer)>

Information website for the freshwater and estuarine harmful algal bloom program – <https://www.waterboards.ca.gov/water_issues/programs/swamp/freshwater_cyanobacteria.html>

Technical report; “California State Water Boards’ framework and strategy for freshwater harmful algal bloom monitoring” – <https://ftp.sccwrp.org/pub/download/DOCUMENTS/TechnicalReports/1141_FHABStrategy_FullReport.pdf>

News story; “Blue-green algae poisoning to blame for death of dogs, pet parents beware” – <https://www.cbs58.com/news/blue-green-algae-poisoning-to-blame-for-death-of-dogs-pet-parents-beware>

**Uruguay**

Scientific article; “Cyanobacterial bloom monitoring and assessment in Latin America” – <https://doi.org/10.1016/j.hal.2023.102429>

Interactive maps for the identification of algal blooms (in Spanish) – <https://www.ambiente.gub.uy/oan/geoportal/identificacion-de-floraciones-algales-2/>

Interactive map of beach conditions in Montevideo (in Spanish) – <https://montevideo.gub.uy/estado-de-las-playas-de-montevideo>

Information on beaches in Montevideo (in Spanish) – <https://montevideo.gub.uy/playas>

**Supplementary Material S12:** Summary of responses to Question 28 related to risk management interventions adopted at the location of the respondent.

| **Country** | **Waterbody closures** | **Public health advisories** | **Cyanobacteria removal / reduction strategies implemented** | **Limit high-exposure activities** | **Stop water sport events** | **Limit promotion as a bathing site** | **Depends on region** | **Don’t know** | **None** |
| --- | --- | --- | --- | --- | --- | --- | --- | --- | --- |
| Argentina |  |  |  |  |  |  | 1 |  |  |
| Australia | 2 | 1 |  |  |  |  |  |  |  |
| Belgium | 3 | 1 | 1 |  |  |  |  |  |  |
| Brazil |  |  |  |  |  |  |  | 1 | 1 |
| Canada | 7 | 4 |  |  |  |  | 1 | 1 | 4 |
| Chile |  | 1 |  |  |  |  |  |  |  |
| Cyprus | 1 |  |  |  |  |  |  |  |  |
| Ecuador |  |  |  |  |  |  |  |  | 1 |
| Estonia |  |  |  |  |  |  |  |  | 1 |
| Finland |  |  | 1 |  |  |  |  |  | 1 |
| France | 5 | 1 |  | 1 |  |  |  |  |  |
| Germany | 2 | 1 | 1 |  |  |  |  |  |  |
| Greece | 1 |  |  |  |  |  |  |  |  |
| Israel | 1 |  |  |  |  |  |  |  |  |
| Italy | 2 |  |  |  |  |  |  |  |  |
| Japan |  |  | 1 |  |  |  |  |  | 3 |
| Kenya | 1 |  |  |  |  |  |  |  | 1 |
| Macedonia |  |  |  |  |  |  |  |  | 1 |
| Malaysia |  |  |  |  |  |  |  |  | 1 |
| Namibia |  |  |  |  |  |  |  |  | 1 |
| Netherlands | 1 | 2 |  | 1 | 1 | 1 |  |  |  |
| New Zealand |  | 3 |  |  |  |  |  |  |  |
| Norway | 2 |  |  |  |  |  |  |  |  |
| Paraguay |  | 1 |  |  |  |  |  |  |  |
| Poland | 1 |  |  |  |  |  |  |  |  |
| Portugal | 1 |  |  |  |  |  |  |  |  |
| Russia | 1 |  |  |  |  |  |  |  | 1 |
| Slovenia |  |  |  |  |  |  |  |  | 1 |
| South Africa |  |  |  |  |  |  |  |  | 2 |
| South Korea |  |  |  |  |  |  |  |  | 1 |
| Spain | 1 |  |  |  |  |  |  | 1 | 1 |
| Sweeden |  |  |  |  |  |  |  |  | 1 |
| Switzerland | 1 |  |  |  |  |  |  |  |  |
| Thailand |  |  |  |  |  |  |  |  | 1 |
| United Kingdom | 6 |  | 1 | 2 | 1 |  |  |  |  |
| USA | 15 | 4 | 2 | 1 |  |  |  | 1 | 2 |
| Uruguay |  | 2 |  |  |  |  |  |  |  |
| **Total Countries** | **19** | **11** | **6** | **4** | **2** | **1** | **2** | **4** | **18** |

**Supplementary Material S13:** Survey responses to Question 25 relating to trigger values / criteria for issuing public health warnings.

| **Country** | **Survey Response** |
| --- | --- |
| Belgium | If > 20,000 cells/mL or 10 μg/L Chl-a, public warnings are carried out by installing signs on field advising citizen to avoid fishing in the pond, to avoid letting their dog drinking from the water and to avoid any contact with the water. If > 100,000 cells/mL or 50 μg/L Chl-a, the monitoring is carried out even more frequently, the awareness signs are installed on field, protective measures are carried out (water pump activation, make the water circulate, etc.). |
| Belgium | Presence of scum or cyano-chlorophyll (measured with algae torch) > 75 µg/L. |
| Brazil | Primary contact and public supply after simplified treatment: 20,000 cells/mL.  Primary contact and public supply after conventional treatment: 50,000 cells/mL.  Secondary contact, supply after conventional or advanced treatment: 100,000 cel/mL, saxitoxins: 3.0 µg Eq./L, Microcystins: 1.0 µg/L. |
| Brazil | >20,000 cells/mL. |
| Brazil | Whenever the number of cyanobacteria in the water used for management exceeds 20,000 cells/mL, according to P5-MS, monitoring should be carried out weekly at the collection point. However, this number often exceeds 1 million units and monitoring is not always expanded. |
| Canada | Visual presence of shoreline scums confirmed by cell counts > 100,000 cells/mL. |
| Canada | For smaller recreational lakes, the following combination of factors trigger a warning: phycocyanin levels, scums greater than 9 square metres, plus microscopic analysis to determine if a harmful taxa is present. I convey the findings to the cottager's association and they send out a bulk email to local residents advising of the problem location, species detected, and safety precautions. |
| Canada | Whole waterbodies closed based on visual reports. |
| Canada | Shoreline scums are reported and municipalities may send out an advisory to residents once they visit themselves. |
| Canada | Alberta follows Health Canada's recreational water quality guidelines for blooms in recreational water including 50,000 cells/mL for cell counts and 10 µg/L for microcystins. |
| Canada | I believe it can be a count, toxin concentration, or visible bloom that support warnings. |
| Canada | No, there's no firm trigger level. A representative from the local Health Unit will go to the beach (reports are nearly always from beaches) to inspect the potential bloom and take a sample, if they deem necessary. |
| Canada | In line with the Canadian guidelines, cyanobacteria or their toxins (microcystin) must be less than 10 µg/L. |
| Canada | Safety levels for recreational activities are 16 µg/L for microcystin-LR and of 40 µg/L for anatoxin levels. |
| Canada | Confirmed Cyanobacteria presence based on taxonomic ID is assumed to be toxin producing. If located in a publicly an accessible area, an advisory could be issued. |
| Chile | They are generally based on WHO recreational water levels. But basically it is because of the change in the coloration of the water. |
| China | Presence of shoreline scums. |
| Croatia | If the biomass of cyanobacteria is more than 2 mg/L an adjustment of the national index values is required (in freshwaters). |
| Ecuador | Only colour is used as indicator of algal bloom. |
| France | Yes, biovolume higher than 1 mm^3^/L. |
| France | Planktonic: biovolume of toxic cyanobacteria > 1mm^3^/L, presence of scums.  Benthic: % dominance of toxic cyanobacteria (no real threshold). |
| Germany | Warning, especially when biovolume concentration above 10 mm^3^/L. |
| Israel | The triggers for early warning in Lake Kinneret are:  > 25 *Microcystis* colonies/mL or  > 50 filaments of *Chrysosporum* or  > 2,000 filaments of *Raphidiopsis* or  > 10 µg/L of chlorophyll from cyanobacteria in PhytopPAM or floating scums  In those cases, if toxin-producing species are abundant in the water samples, toxin determinations in water and in scums are initiated. |
| Japan | None. |
| Kenya | None. |
| Macedonia | None. |
| Namibia | We use > 20,000 cells/mL as per the RSA aquatic guidelines for our lakes. |
| New Zealand | Yes - amber alert is above 20% cover and red alert is above 50% cover and/or presence of moderate amounts of detached mats. |
| Norway | WHO guidance levels for biovolume and cyanotoxins recreational waters used - using the most recent Chorus & Walker 2021 guidance. Also use visible surface scums (often reported through Bloomin Algae app records) as a trigger for local authorities as these are high risk (as outlined in the Alert level 2 in this guidance). |
| Norway | There are no national legislation for risk evaluation on cyanobacteria in Norway. We are following the WHO framework and guidelines. |
| Paraguay | We have a basic list of parameters for our monitoring activities based on various regulations:  1) Local Regulation (Resolution 222/02): pH, dissolved oxygen, total nitrogen (TN), total phosphorus (TP), and fecal coliforms.  2) International Guidelines (WHO, 2021): Transparency and chlorophyll-a. For cyanobacteria, the WHO recommends measuring biovolume. Since we measure in cells/mL, we apply an adjustment based on a regional publication that established an approximate relationship between biovolume/mL and cells/mL.  Therefore, > 60,000 cells/mL serves as our Level 1 alert for recreational water use. |
| Paraguay | First of all, Paraguay's regulations (MADES) have not yet updated the maximum algal bloom alert values since 2002 (here it is > 200,000 cells/mL, which is much higher). Typically, the community communicates directly to the media by sending videos about the blooms, and based on media publications, authorities tend to respond with monitoring (which does not necessarily include short- or long-term follow-up or management). |
| Poland | After visually detecting blooms, bathing areas are closed. |
| Portugal | A national consensus is still in development but WHO guidelines are being considered. At this stage, >20,000 cells/mL and MC > 10 µg/L is sufficient. |
| Slovenia | None. |
| South Africa | None. |
| South Africa | None. |
| South Korea | Attention Level: When the cyanobacterial cell count is between 1,000 and 10,000 cells/mL in two consecutive samples.  Caution Level: When the cyanobacterial cell count is between 10,000 and 1,000,000 cells/mL in two consecutive samples.  Algal Bloom: When the cyanobacterial cell count is 1,000,000 cells/mL or higher in two consecutive samples.  Lifting of Alert: When the cyanobacterial cell count is below 1,000 cells/mL in two consecutive samples. |
| Spain | >20,000 cells/mL according to Chorus et al. (WHO, 2021). |
| Spain | None. |
| Sweeden | Only visible bloom. |
| Switzerland | When scums are reported (mostly by citizens) we take a sample, hava a look at in under the microscopy/send it to a plankton expert, if necessary send it for toxin analysis, if it is toxic or has the potential to be toxic, we inform the municipality, the municipality is responsible to close the shore/lake access and to inform the people with a poster. |
| United Kingdom | As far as I know, standard WHO guidelines are followed (Chorus and Walker). |
| United Kingdom | We now report against the most recent WHO guidance using biovolume concentration of cyanobacteria. |
| United Kingdom | WHO guidelines. |
| USA | Green water by visual assessment (nothing quantitative). |
| USA | Unsure but probably WHO cell numbers and cyanotoxin concentrations; i.e., microcystin 8 µg/L, cylindrospermopsin 15 µg/L. |
| USA | Microcystin detection by Abraxas test strips. |
| USA | In Oregon, our recreational trigger is over 8 ppb for microcystin and 15 ppb for cylindrospermopsin. As we approach those values, we reach out to the state to possibly declare an advisory. |
| USA | Following Pennsylvania DEP Guidelines:  Cyanobacteria colony density: 300 natural units = Advisory, 1500 natural units = Avoid Primary Contact  Microcystin: <8 µg/L = Advisory, 8 µg/L = Avoid Primary Contact  Cylindrospermopsin: <15 µg/L = Advisory, 15 µg/L= Avoid Primary Contact  Anatoxin-a (provisional): <15 µg/L = Advisory, 15 µg/L = Avoid Primary Contact  Saxitoxin (provisional): <8 µg/L = Advisory, 8 µg/L = Avoid Primary Contact |
| USA | >70,000 cells/mL. |
| USA | If detached benthic mat samples return cyanotoxin concentrations above any Washington Department of Health (WADOH) recreational guidelines, we communicate the results with the Natural Resource Management team who will alert local authorities associated with the water body. Boat ramp and swim beach closures are considered, based on WADOH guidance. Signage is posted and our Public Affairs Office sends out press releases. |
| USA | Currently Tennessee does not have numeric criteria for public warnings for cyanobacteria. We use a narrative criteria similar to what Florida has. Visible scum or mat with PTOX cyanobacterial genera present would issue a Precautionary advisory. Detection of cyanotoxin (any concentration) would issue a Warning Advisory. |
| USA | LOW THRESHOLD (warning signs are posted); Microcystins: > 8 µg/L, Anatoxin-a: > 1 µg/L.  HIGH THRESHOLD (danger signs posted & parks departments generally close access points to water); Microcystins: > 2,000 µg /L, Anatoxin-a: > 300 µg/L. |
| USA | We use EPA values for microcystins (8 ug/L) and cylindrospermopsin (15 ug/L) to trigger an advisory. We will put an advisory out without toxin data is visually we have any suspicion it could be a HAB. |
| USA | No specific triggers, we just give general warnings about relative risks. |
| USA | Presence of green water or shoreline scums may be used as justification to close a public beach. |
| USA | Health alert issued when detection of cyanotoxin above laboratory MDL (e.g. ≥ 0.25 µg/L total microcystins). |
| USA | Advisory trigger values; > 8 µg/L microcystin; > 15 µg/L cylindrospermopsins; > 8 µg/L saxitoxin; > 15 µg/L anatoxin-a. |
